# A generative network model of neurodevelopmental diversity in structural brain organization

**DOI:** 10.1038/s41467-021-24430-z

**Published:** 2021-07-09

**Authors:** Danyal Akarca, Petra E. Vértes, Edward T. Bullmore, the CALM team, Kate Baker, Susan E. Gathercole, Joni Holmes, Rogier A. Kievit, Tom Manly, Joe Bathelt, Marc Bennett, Giacomo Bignardi, Sarah Bishop, Erica Bottacin, Lara Bridge, Diandra Brkic, Annie Bryant, Sally Butterfield, Elizabeth M. Byrne, Gemma Crickmore, Edwin S. Dalmaijer, Fánchea Daly, Tina Emery, Laura Forde, Grace Franckel, Delia Fuhrmann, Andrew Gadie, Sara Gharooni, Jacalyn Guy, Erin Hawkins, Agnieszka Jaroslawska, Sara Joeghan, Amy Johnson, Jonathan Jones, Silvana Mareva, Elise Ng-Cordell, Sinead O’Brien, Cliodhna O’Leary, Joseph P. Rennie, Ivan Simpson-Kent, Roma Siugzdaite, Tess A. Smith, Stephani Uh, Maria Vedechkina, Francesca Woolgar, Natalia Zdorovtsova, Mengya Zhang, Duncan E. Astle

**Affiliations:** 1 MRC Cognition and Brain Sciences Unit, University of Cambridge, Cambridge, UK; 2 Department of Psychiatry, University of Cambridge, Cambridge, UK; 3 The Alan Turing Institute, London, UK; 4 Department of Clinical Neurosciences, Wolfson Brain Imaging Centre, University of Cambridge, Cambridge, UK

**Keywords:** Network models, Neuronal development, Dynamic networks, Neurodevelopmental disorders

## Abstract

The formation of large-scale brain networks, and their continual refinement, represent crucial developmental processes that can drive individual differences in cognition and which are associated with multiple neurodevelopmental conditions. But how does this organization arise, and what mechanisms drive diversity in organization? We use generative network modeling to provide a computational framework for understanding neurodevelopmental diversity. Within this framework macroscopic brain organization, complete with spatial embedding of its organization, is an emergent property of a generative wiring equation that optimizes its connectivity by renegotiating its biological costs and topological values continuously over time. The rules that govern these iterative wiring properties are controlled by a set of tightly framed parameters, with subtle differences in these parameters steering network growth towards different neurodiverse outcomes. Regional expression of genes associated with the simulations converge on biological processes and cellular components predominantly involved in synaptic signaling, neuronal projection, catabolic intracellular processes and protein transport. Together, this provides a unifying computational framework for conceptualizing the mechanisms and diversity in neurodevelopment, capable of integrating different levels of analysis—from genes to cognition.

## Abstract (web summary)

The formation of large-scale brain networks represents crucial developmental processes that can drive individual differences in cognition and which are associated with multiple neurodevelopmental conditions. Here, the authors use generative network modelling to provide a computational framework for understanding neurodevelopmental diversity.

## Introduction

The human brain is highly organized at multiple scales. At the broadest scale, neuronal populations are structurally connected across large anatomical distances with white-matter fiber bundles, forming a set of interconnected networks. This macroscopic organization can be studied via diffusion-weighted magnetic resonance imaging (MRI), which measures the direction of water diffusion in vivo^1–3^. During childhood the emergence and continual refinement of these large-scale brain networks allows for increasing functional integration and specialization^4,5^. This process is thought crucial for the growth of complex cognitive processes such as language^6^ and executive function^7–12^. However, there are individual differences in the organization of these networks across children, and these differences mirror important developmental outcomes. Indeed, differences in macroscopic networks have been implicated across multiple neurodevelopmental conditions^13^, including ADHD^14^, autism^15,16^, and language disorders^17^.

But what mechanisms drive the diversity of macroscopic brain networks? And how do these mechanisms give rise to individual differences in children’s outcomes? There are numerous descriptive theories^18–22^ that speculate about how different levels of analysis (e.g., genes, brain structure, and function) interact to produce these neurodevelopmental differences. However, to date no theories are sufficiently specified that they can simulate individual-level brain networks. In the absence of computational models, it is difficult to establish mechanistic links between individual differences in observations (e.g., gene expression, biological pathways, system wide organization). This theory gap represents a major limitation for understanding neurodevelopmental diversity. The purpose of this study is to address precisely this gap, by modeling the generative wiring properties of a large sample of children at heightened neurodevelopmental risk of poor outcomes. The computational model we implemented is guided by a simple principle: the brain’s structural organization is shaped by an economic trade-off between minimizing wiring costs and adaptively enhancing valuable topological features^23^. We hypothesize that the emergence of whole-brain organization reflects the continual trade-off of these factors over time and that tiny differences in the parameters governing the trade-off can produce the neurodiverse outcomes we observe. Somewhat counterintuitively, tight parameter constraints likely enable macroscopic neurodiversity, because large changes in these parameters would produce networks with configurational states that are not observed in reality. Instead, narrow boundaries reflect parameter conditions within which networks can be different, but still maintain adequate structural properties to be functional.

Our work utilizes generative network modeling^24,25^, in which connections within a physically embedded network are formed probabilistically over time according to a wide range of potential mathematical constraints. Varying the parameters and wiring rules that govern network formation provides a way of establishing which statistics likely create real networks—in this case structural brain networks in our large heterogeneous sample of children. Specifically, we: (1) tested which topological features should be valued in the wiring trade-off to produce highly accurate individual child connectomes; (2) tested how small changes in these parameters alter the organizational properties of the resulting networks; (3) established relationships between these different wiring parameters and cognitive outcomes; (4) identified genes with expression profiles that were spatially co-located with those topological features; and (5) established the biological pathways that are enriched in these gene lists. Together, this provides a computational framework that mathematically specifies the formation of a network over time, captures individual differences in brain organization and cognition, and incorporates the genetic and biological pathways that likely constrain network formation.

## Results

### The generative network model

**Fig. 1:**
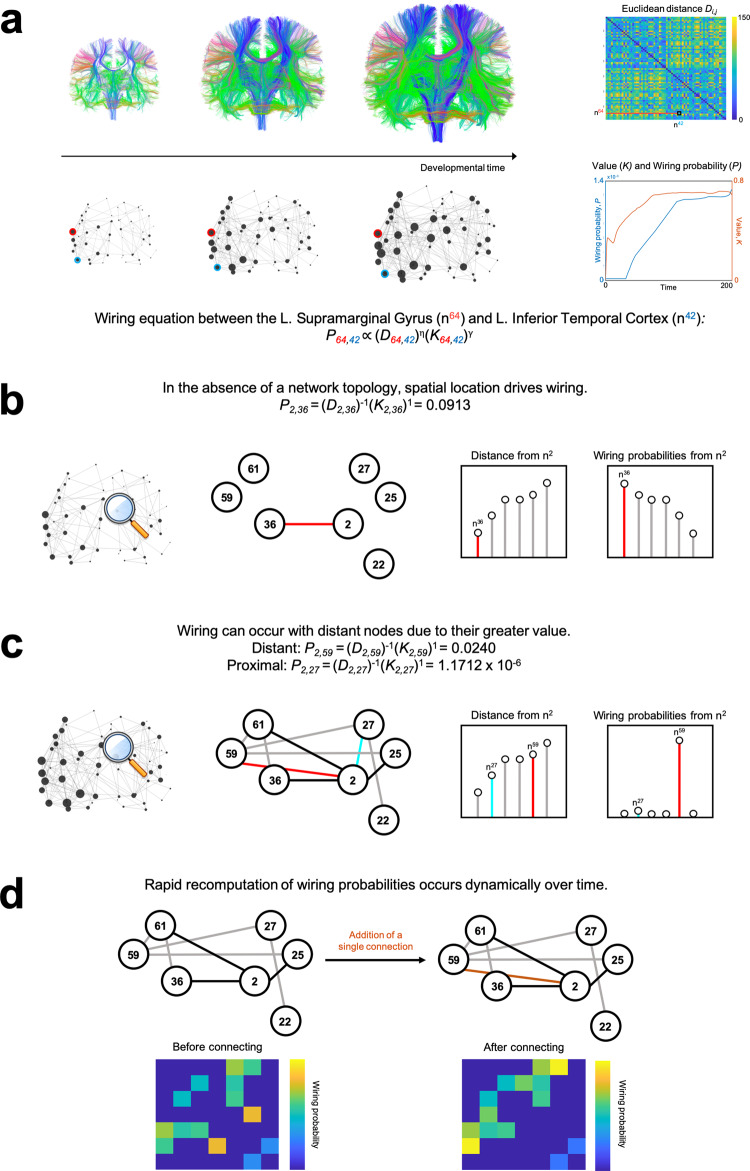
Updating wiring probabilities within the generative network model iteratively, based on dynamically changing graphical structures. **a** The brain’s structural connectivity is modeled as a generative network which grows over time according to parametrized connection costs, (*D*_*i,j*_)^*η*^ and values, (*K*_*i,j*_)^*γ*^. In this illustration, we use subject one’s optimal model. **b** Early in network development, the absence of a topology leads to proximal nodes being much more likely to form connections. The displayed distances and probabilities are from the right caudal anterior cingulate (*n*^2^), which corresponds to row (*D*_2_,:)^*η*^ and (*P*_2_,:). We display it’s six nearest cortical regions. **c** Later, the relative values (*K*_*i,j*_) between nodes influence connection probabilities, such that nodes which are more distant (e.g., left rostral anterior cingulate, *n*^59^ in red) may be preferred to nodes which are closer (e.g., right superior frontal cortex, *n*^27^ in cyan). **d** As costs and values are decoupled, the wiring probability can be rapidly recomputed when dynamic changes in graphical structure occur over developmental time.

The generative network model (GNM) can be expressed as a simple wiring equation^24,25^ (Fig. 1a). If you imagine a series of locations within the brain, at each moment in time the wiring equation calculates which two locations will become connected. It calculates this wiring probability by trading-off the cost of a connection forming, against the potential value of the connection being formed. The equation can be expressed as:

1 $${P}_{i,j}\propto {({D}_{i,j})}^{\eta }{({K}_{i,j})}^{\gamma },$$

where *D*_*i,j*_ represents the Euclidean distance between nodes *i* and *j* (i.e., “costs”), and *K*_*i,j*_ reflects the value (i.e., “attractiveness”) in forming a connection. *P*_*i,j*_ represents the wiring probability as a function of the product of the parameterized costs and value. The end result is a wiring probability matrix which updates over time as new connections are added.

*D*_*i,j*_ is parameterized by the scalar *η*, which changes how node-to-node distances influence their probability of connecting. For example, when *η* is negative, wiring probabilities decline when distances increase, and this reflects the costliness of forming connections with nodes that are distant. This is traded-off against *K*_*i,j*_, which represents some relationship between nodes, which can be thought of as a topological value (or “rule”) driving the intention for node *i* to connect with node *j*. *K*_*i,j*_ is parameterized by a distinct scalar *γ*. *K*_*i,j*_ can take a range of different forms and can, in principle, be selected from any non-geometric growth rule used to model social and economic networks^26–28^. One simple example is the “matching” rule^24^: nodes form connections with other nodes on the basis of their normalized overlap in neighborhood—i.e., whether nodes are connected to similar nodes to themselves (also termed homophily).

To make this more concrete, imagine the following scenario: a network is growing according to the matching rule, preferentially attaching to nodes which are both similarly connected and spatially proximal. In the wiring equation, this would be represented as *η* being negative (e.g., *η* = −1), *K*_*i,j*_ represented as normalized neighborhoods between nodes (i.e., matching) and its parameter *γ* being positive (e.g., *γ* = 1). In short, a node being far away makes it less likely that a new connection will be formed, but it having a similar a neighborhood increases the likelihood. Suppose that the right caudal anterior cingulate (Node 2, *n*^2^) is going to wire to one of its six nearest neighbors. Initially, due to an absent network topology, spatial proximity has a great influence in the formation of new connections—it will wire to its nearest neighbor (Fig. 1b). However, gradually over time, the network’s developing structural topology means that *K*_*i,j*_ (i.e., the relationships between nodes) may now have a greater influence on wiring probabilities. Indeed, the right caudal anterior cingulate may later wire with a node that, although further away than other available nodes, has a greater value (i.e., matching) than the others (Fig. 1c). As the wiring equation separately parameterize costs and value, the presence of a single connection can heavily influence the topology of the network and thus the future updated wiring probabilities. This is because new connections can lead to entirely new overlapping neighbors, which may include distant nodes. As a result, wiring probabilities can change considerably from moment to moment, despite costs remaining fixed (Fig. 1d).

The GNM simulates this process across the whole brain, until the overall number of connections matches those found in the observed brain network. Subsequently, to test the accuracy of the simulation, an energy function, *E*, must be defined which measures the dissimilarity between simulated and observed networks^24,25^:

2 $$E={\rm{max}}({\rm{KS}}_{k},{\rm{KS}}_{c},{\rm{KS}}_{b},{\rm{KS}}_{e}),$$

where KS is the Kolmogorov–Smirnov statistic comparing degree *k*, clustering coefficient *c*, betweenness centrality *b*, and edge length *e* distributions of simulated and observed networks. Minimizing *E* finds parameters *η* and *γ* which generate networks most closely approximating the observed network.

The four measures in the energy equation are good candidates for evaluating the plausibility of simulated networks. They are critical statistical properties of realistic networks and have featured within the most well-documented simulated network models^29–31^. Moreover, these statistical properties have been implicated in a number of neuropsychiatric conditions^32,33^ in addition to being shown to be heritable^34^.

### Small variations in GNM parameter combinations produce accurate and spatially embedded networks

**Fig. 2:**
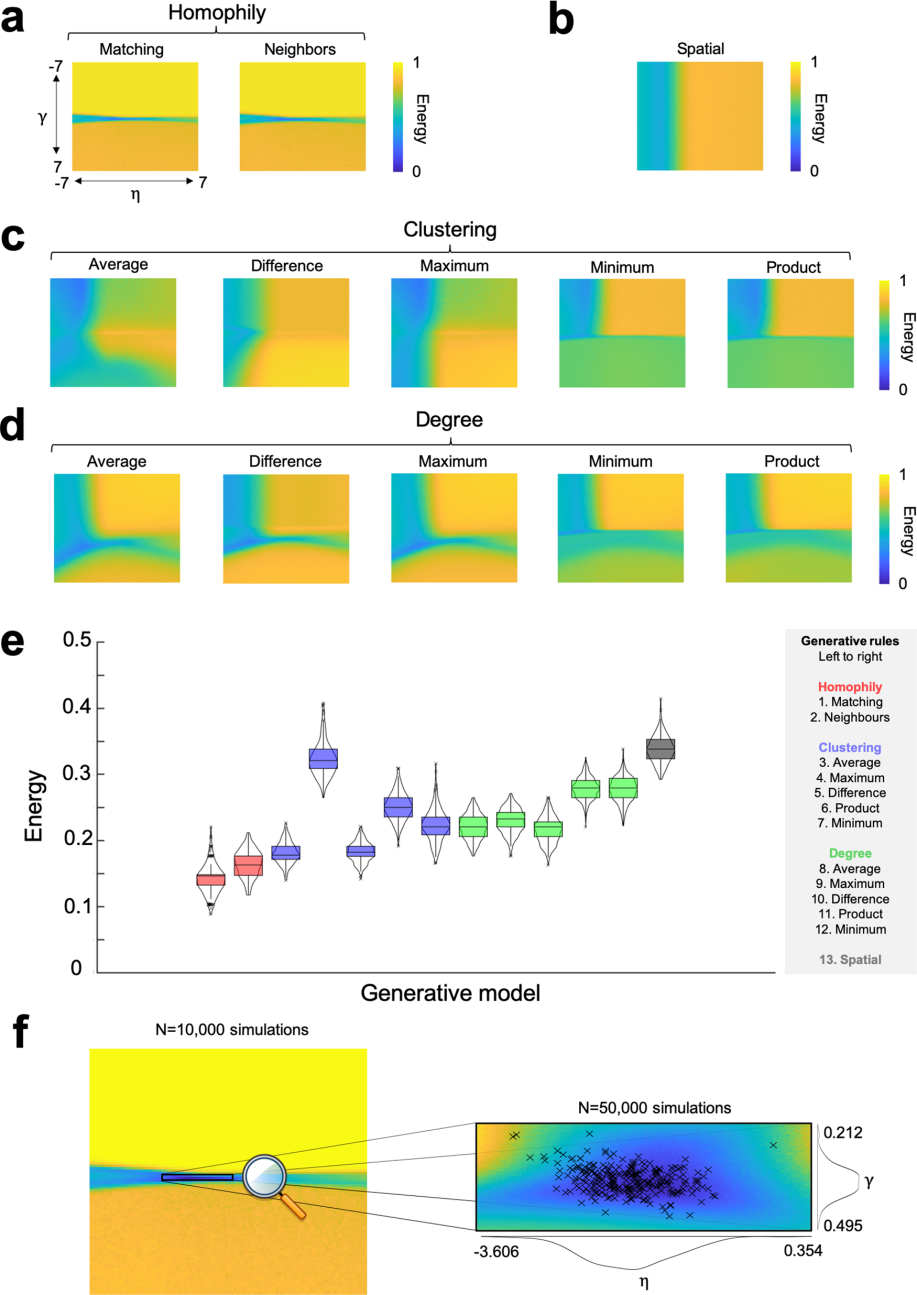
Sample-averaged energy landscape visualization and generative rule comparisons. **a** Homophily-based methods. Matching and neighbors algorithms calculate a measure of shared neighborhood between nodes. **b** The spatial method. This ignores *γ* entirely, judging networks only on the basis of their spatial relationship. **c** Clustering-based methods. These calculate a summary measure between two nodes in terms of their clustering coefficients. **d** Degree-based methods. These calculate a summary measure between two nodes in terms of their degree. **e** Energy statistics from the best performing simulation across 13 generative rules, showing that matching can achieve the lowest energy networks given the appropriate parameter combination. In total, there are *N* = 270 data points for each of the 13 boxplots. A tabulated form of this figure is provided in Supplementary Table 1. The boxplot presents the median and IQR. Outliers are demarcated as small black crosses, and are those which exceed 1.5 times the interquartile range away from the top or bottom of the box. **f** A further 50,000 simulations were undertaken in the refined matching window, as these defined boundary conditions for which low-energetic networks were consistently achieved. Each cross represents a subject’s individually specific wiring parameters that achieved their lowest energy simulated network.

From a basic seed network common to all participants (for detail, see Supplementary Fig. 1 and Methods), we computed the subject-wise optimal GNM (i.e., network with lowest energy) over a range of 10,000 evenly spaced parameter combinations (−7 ≤ *η* ≤ 7, −7 ≤ *γ* ≤ 7) using 13 different generative rules (for rule formulae, see Supplementary Table 1) across our large sample of children (*N* = 270, 178 males, 92 females, mean age = 9 years 10 months, SD age = 2 years 2 months; full sample details can be found at Holmes et al.^35^). In each case, we computed energy landscapes to contextualize how they perform (Fig. 2a–d). Mirroring findings in adult samples^24,25,35^, we found that models driven by geometry and topology outperform the pure geometric spatial model and homophily-based models achieve the lowest energy for our pediatric sample (Fig. 2e). In other words, when one combines the distance penalty with the “matching rule” we described in our concrete example (as shown in Fig. 1), it produces the most plausible simulated brain networks. This difference between generative rules is extremely robust. A post hoc power calculation revealed that the homophily-based rules could be distinguished from the next best class with near-perfect statistical power (*t* = −10.210, *p* = 6.705 × 10^−21^; *N* = 270, power > 0.99), and that this difference could be detected with around 70 participants.

It is notable that across the matching energy landscape, these plausible networks exist within an extremely narrow window of parameter solutions. That is, as a proportion of the parameter space, the matching rule (and the other homophily-based model “neighbors”) contain the least number of low-energy networks relative to other rules. But as Fig. 2e shows, these networks are the closest to real networks. Thus, varying homophily-based parameters produces the most realistic networks, yet has the lowest variability in the space (Supplementary Fig. 2).

While small, variability within this narrow matching window determines inter-individual differences in brain network growth. This is because small changes in parameters (i.e., the magnitude and direction in which costs and values influence wiring probabilities) can lead to networks which are diverse yet include basic structural properties common to all subjects. To derive more precise estimations of optimal generative parameter combinations, we subsequently generated a new set of 50,000 evenly spaced simulated networks over this narrow low-energy matching window (−3.606 ≤ *η* ≤ 0.354, 0.212 ≤ *γ* ≤ 0.495). Focusing on this energy crevasse allows us to detect individual differences in optimal parameter combinations with much greater specificity. In other words, we resampled the parameter combinations focusing within the low-energy window, to make sure we have the most precise estimate of each individual child’s optimal parameters.

In Fig. 2f, we show the spatial distribution of these top performing parameter combinations and Supplementary Table 2 documents their summary statistics. These finely calibrated networks are even more low energy than in the previous analysis. In Supplementary Fig. 3a–d we detail how KS statistics vary across the same space. Importantly, due to the stochastic nature of GNMs, the energy of optimal parameter combinations varies with an average standard deviation (SD) of 0.045 across the sample (1000 independent runs). Therefore, for the rest of this study, we quote our parameter analyses averaged across a variable number of wiring parameters which achieved networks with the lowest energy in the space: *N* = 1 (equating to 0.002% of the space) *N* = 10 (0.02%), *N* = 100 (0.2%), and *N* = 500 (1.0%).

The optimal *η* and *γ* parameters are significantly negatively correlated with each other, such that subjects with large *γ* parameters tend to have larger negative *η* (Best *N* = 1 network: *r* = −0.284, *p* = 2.07 × 10^−6^; *N* = 10 networks: *r* = −0.403, *p* = 6.08 × 10^−12^; *r* = −0.460, *p* = 1.58 × 10^−15^; *N* = 100 networks: *p* = −0.460, *p* = 1.58 × 10^−15^, *N* = 500 networks: *r* = −0.497, *p* = 3.21 × 10^−18^) (Supplementary Fig. 3f). Optimally simulated networks, using this simple wiring equation, are so similar to the actual networks that a support vector machine is unable to distinguish them using the parameters from the energy Eq. (2) (mean accuracy = 50.45%, SD = 2.85%).

Replicating previous work, we find that our simulated networks, optimized via the statistical properties included in the energy Eq. (2) via homophily generative mechanisms, accurately capture these properties in observed networks^24,25,36^. But do these capture crucial network properties not included in the energy equation, like their spatial embedding? We next examined if the spatial patterning of these network properties arises simply from the generative model.

**Fig. 3:**
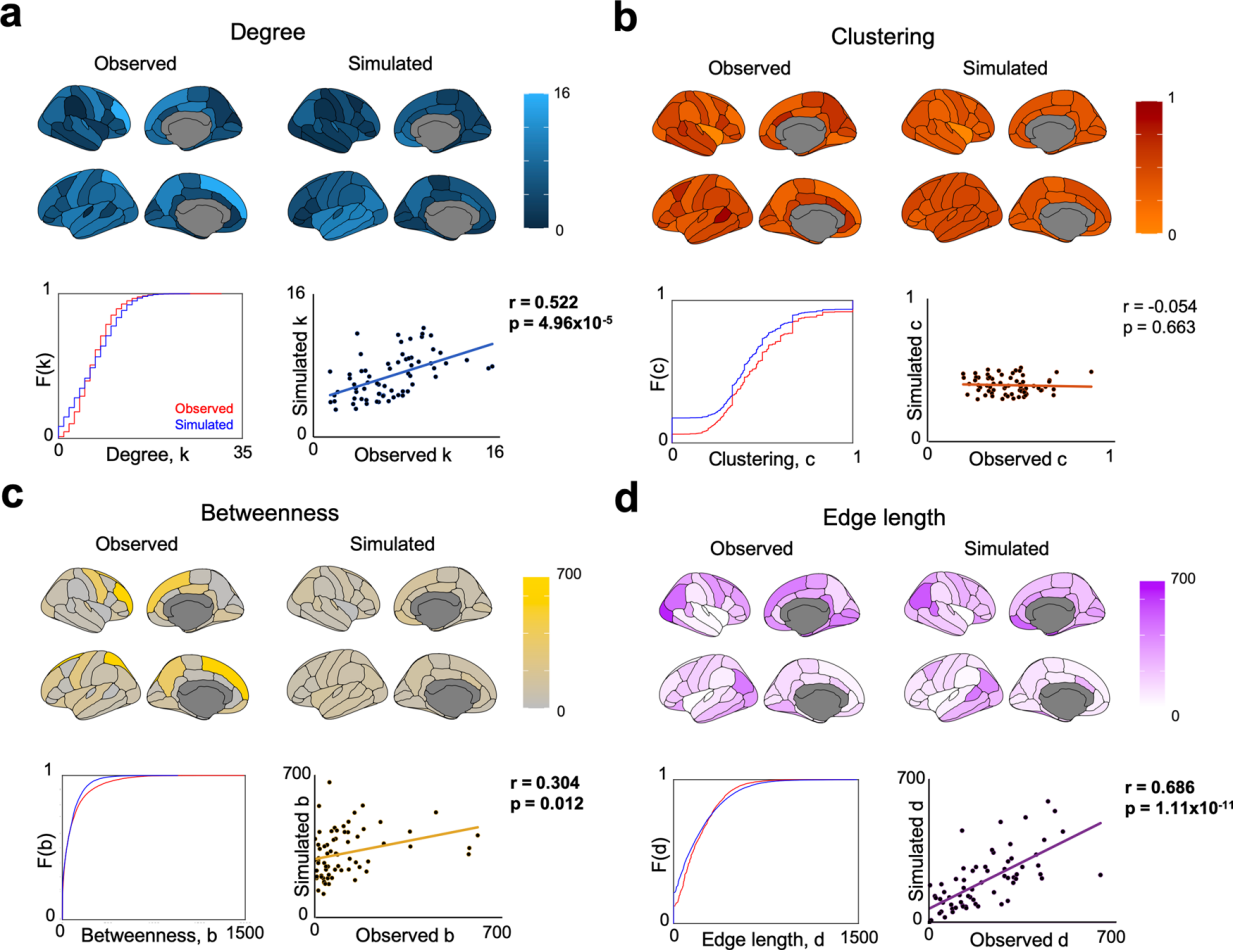
Spatial embedding of simulated networks grown via optimized homophily generative mechanisms. For each network measure, we present the cumulative density functions across all observed versus simulated nodes within each network. Each point in the scatter plot shows one of the 68 across-subject average nodal measures from the observed and optimally simulated networks. We also show a visualization of these measures. All statistics were computed via two-tailed linear correlations, quoting the Pearson’s correlation coefficient. **a** Degree between observed and simulations are significantly positively correlated (*r* = 0.522, *p* = 4.96 × 10^−5^). **b** Clustering between observed and simulations at not correlated (*r* = −0.054, *p* = 0.663). **c** Betweenness centrality between observed and simulations are significantly positively correlated (*r* = 0.304, *p* = 0.012). **d** Edge length (as a summation of all edges from each node) between observed and simulations are significantly positively correlated (*r* = 0.686, *p* = 1.11 × 10^−11^). Boldened values are significant correlations at *p* < 0.05. In Supplementary Fig. 4, we present a parallel analysis including local and global measures not included in the energy equation. In Supplementary Fig. 5, we demarcate for each measure the generative error in spatial embedding, and show the ranked performance for each region.

Averaged across the sample, optimally performing generative models (i.e., those using the “matching” rule) produce networks which significantly correlate with observed networks in terms of their degree (*r* = 0.522, *p* = 4.96 × 10^−5^), edge length (*r* = 0.686, *p* = 1.11 × 10^−11^), and betweenness centrality (*r* = 0.304, *p* = 0.012) but not clustering coefficient (*r* = −0.054, *p* = 0.663) (Fig. 3). That is, the spatial embedding of these network properties seemingly emerges, to mirror those of the observed networks, despite this not being specified in the growth process. We extended this analysis to new measures outside of the energy equation (Supplementary Fig. 4). While local efficiency and assortativity cannot be significantly predicted across the sample (*r* = 0.211, *p* = 0.084 and *r* = −0.096, *p* = 0.116, respectively), optimally performing simulated and observed networks correlate positively in terms of their global number of rich clubs (*r* = 0.316, *p* = 1.11 × 10^−7^), maximized modularity (*r* = 0.349, *p* = 3.84 × 10^−9^), and transitivity scalar (*r* = 0.411, *p* = 2.11 × 10^−12^). In short, despite not being specified in the growth process, the simple homophily rule generates many properties of observed brain networks.

One criticism of our simulations is that their embedding may be an artifact of the seed network (which is on average 10.8% the density of the observed/simulated networks). In short, if by chance the seed network mirrors the final network, it could be inevitable that spatial embedding would emerge, in terms of node degree, betweenness centrality and edge length. If so, one would expect initial local statistical properties of the seed to be significantly associated with regional accuracy of the simulation. To determine if this is the case, we analyzed the regional accuracy of our homophily simulations by determining their regional generative error (as a mismatch between observed and simulated outcome, depicted in Supplementary Fig. 5). Importantly, the average ranked error (Supplementary Fig. 5e) is not correlated with the seed network’s connectivity (*r* = −0.0711, *p* = 0.5643). Furthermore, seed features do not correlate with their own feature’s resultant error (Degree, *r* = −0.0408, *p* = 0.7410; Betweenness, *r* = 0.1833, *p* = 0.1345; Edge length, *r* = 0.1114, *p* = 0.3659).

The large heterogenous sample we chose is ideal for this computational approach to understanding diversity, but it is highly likely that this approach will work for more standard typically developing cohorts of children. In Supplementary Figure 6 we replicate our key findings in an independent sample of *N* = 140 children recruited from local primary schools in the same area (for more details about the cohort, see “Methods”; “Participants” and Johnson et al.^37^).

### Individual differences in wiring parameters mirror connectome organization, gray matter morphology, and cognitive scores

A critical benefit of a generative modeling approach is that it allows us to probe the underlying mechanisms occurring over the development of the network^38^. As one explicitly specifies the generative mechanisms involved, the statistical properties which “fall out” of the network can be considered as spandrels^39^; epiphenomena of the network’s development according to the much simpler economical trade-off (in this case, according to the homophily principle). If the generative model is indeed capturing biologically relevant processes, one would expect the wiring equation—at a minimum—to reduce the network’s dimensionality simply into the two wiring parameters used to construct it. Under this view, individual differences in wiring parameters should (1) map to wide-ranging statistical properties of the observed network, which may be considered spandrels and (2) appropriately reduce dimensionality of the connectome such that, for example, one can equivalently predict cognitive scores from parameters as one would be able to from the network properties.

**Fig. 4:**
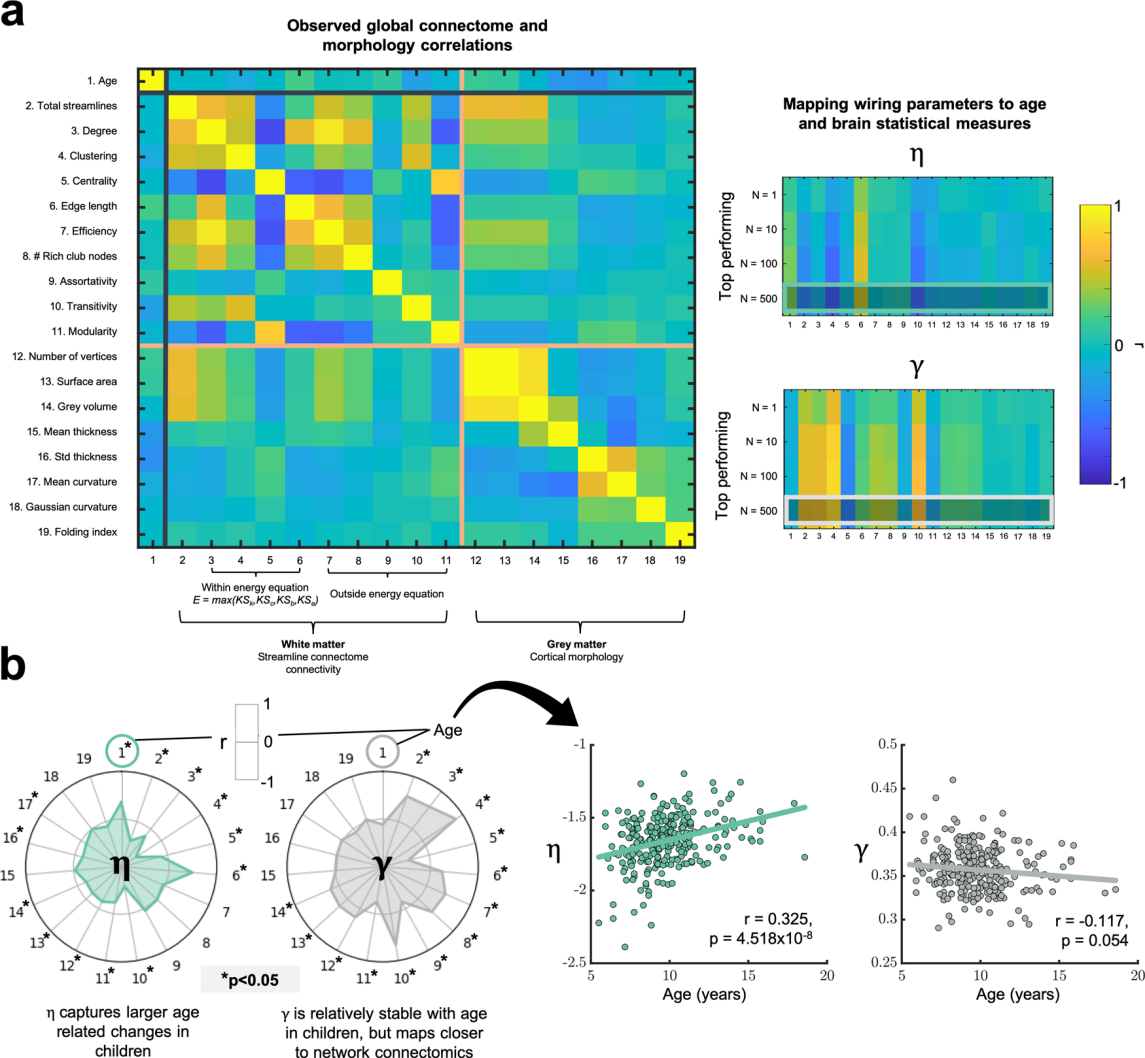
Statistical properties of the connectome and cortical morphology, and their relationships with wiring parameters and age. **a** The correlation matrix of connectome and morphological findings show how each measure correlates with every other measure. Measures 3–6 were included in the energy equation. Measures 7–11 are connectome measures not included in the energy equation. Measures 12–19 are cortical morphological measures. *η* and *γ* are each significantly correlated with a range of measures, both inside and outside of the energy equation. Correlation coefficient matrices are shown, the bottom row of which is highlighted and is reflected in the above radar plots (middle), in addition to the significance matrix (bottom), across varying numbers of top performing parameters, for each of the 19 measures investigated. **b** Radar plots depict the correlations between all measures and *η* (left) and *γ* (right) averaged across the top *N* = 500 parameters in the parameter space. All statistics were computed via two-tailed linear correlations, quoting the Pearson’s correlation coefficient. The asterisk, *, reflects significant correlations at *p* < 0.05. Note, the inner edge of the radar plot reflects negative correlations and the outer edge reflects positive correlations. Specific results for variable top performing parameters are provided in Supplementary Table 3. Further scatter plots are provided highlighting the relationship of wiring parameters with age. *η* has a significantly positive relationship with age (*r* = 0.325, *p* = 4.518 × 10^−8^) while *γ* has a weak non-significant negative relationship with age (*r* = −0.117, *p* = 0.054).

To explore this, we first examined how wiring parameters reflect observed features of brain organization by quantifying how a subject’s *η* and *γ* relate to global measures of their observed connectome. Furthermore, for all 270 subjects, cortical morphology data were available. In Fig. 4, we document how global network and morphological measures (most of which are not included in the energy equation) relate to each other, in addition to their reasonably stable association with a varying number of high performing *η* and *γ* wiring parameters (specific results are provided in Supplementary Table 3), and their associations with age. Figure 4b shows that *η* is significantly associated with age—the network parameters needed to form optimal networks over time need to favor longer distance connections for older participants, relative to younger participants^25^. To disentangle age-related parameter differences from individual differences, we repeated all of our correlations across measures whilst partialling out age (Supplementary Fig. 7). Associations remain when age has been controlled for, demonstrating that age-related changes in optimal parameters are relatively independent of the individual differences in those parameters. This is not only an important step in demonstrating that these parameters generalize to distinct measures (e.g., morphological observations) not used to train the generative models, but also demonstrates that the generative approach is consistent with the notion that wiring parameters themselves have significant associations with numerous statistical properties in a network.

Next, we tested the ability of the wiring equation to reduce the dimensionality of the connectome. Specifically, if wiring parameters are accurate decompositions of an individual’s structural network they should predict cognitive outcomes equivocally to observed features of the connectome. For all 270 subjects we had data from a battery of cognitive tasks, including measures of executive function, phonological awareness, working memory, fluid reasoning and vocabulary (for details of the tasks see “Methods”; “Cognitive and learning assessments”).

**Fig. 5:**
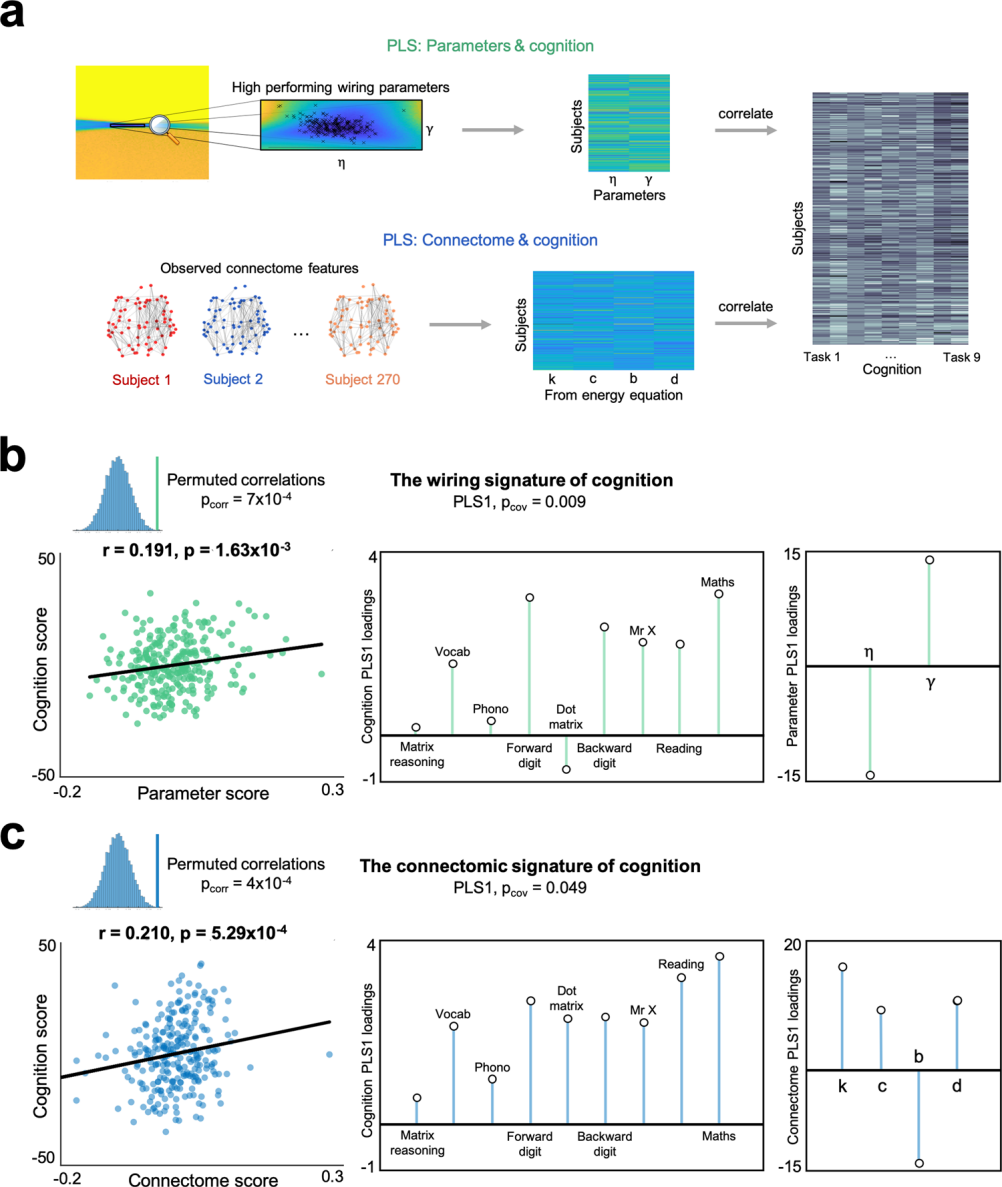
Covarying patterns of wiring parameters and connectome features with cognitive performance across nine cognitive tasks. **a** A visual representation of the two PLS analyses undertaken. **b** There is a significant positive correlation (two-tailed linear correlation, quoting the Pearson’s correlation coefficient) between parameter scores and PLS-derived cognitive scores. PLS1 was statistically significant (*p*_corr_ = 7 × 10^−4^ and *p*_corr_ = 4 × 10^−4^, respectively) for both analyses using *n* = 10,000 permutations. Each parameter loads with similar magnitude onto PLS1. **c** There is an analogous significant positive correlation between connectome scores and PLS-derived cognition scores, using the same statistical procedure.

We tested the relationship between a subject’s age-standardized cognition and (1) their optimal wiring parameters and (2) global measures of their structural connectome (using measures included in the energy equation). This was done using partial least squares (PLS), a multivariate statistical technique which extracts optimally covarying patterns from two data domains^40^. We undertook two separate PLS analyses, which correlated (1) optimal wiring parameter combinations or (2) global connectome measures across our sample, with cognitive performance in the nine tasks, respectively (Fig. 5a). For both analyses, PLS1 was significant in the amount of explained covariance (*p*_cov_ = 0.009 and *p*_cov_ = 0.049, respectively). PLS1 score predictions, and their cognitive loadings, are extremely similar between wiring parameters and connectome features (*r* = 0.191, *p* = 1.63 × 10^−3^, *p*_corr_ = 7 × 10^−4^ and *r* = 0.210, *p* = 5.29 × 10^−4^; each *p*_corr_ = 7 × 10^−4^ and *p*_corr_ 4 × 10^−4^, respectively, from 10,000 permutations of scores) (Fig. 5b, c).

### Variability in neurodevelopmental trajectories arises through value-updating over time

**Fig. 6:**
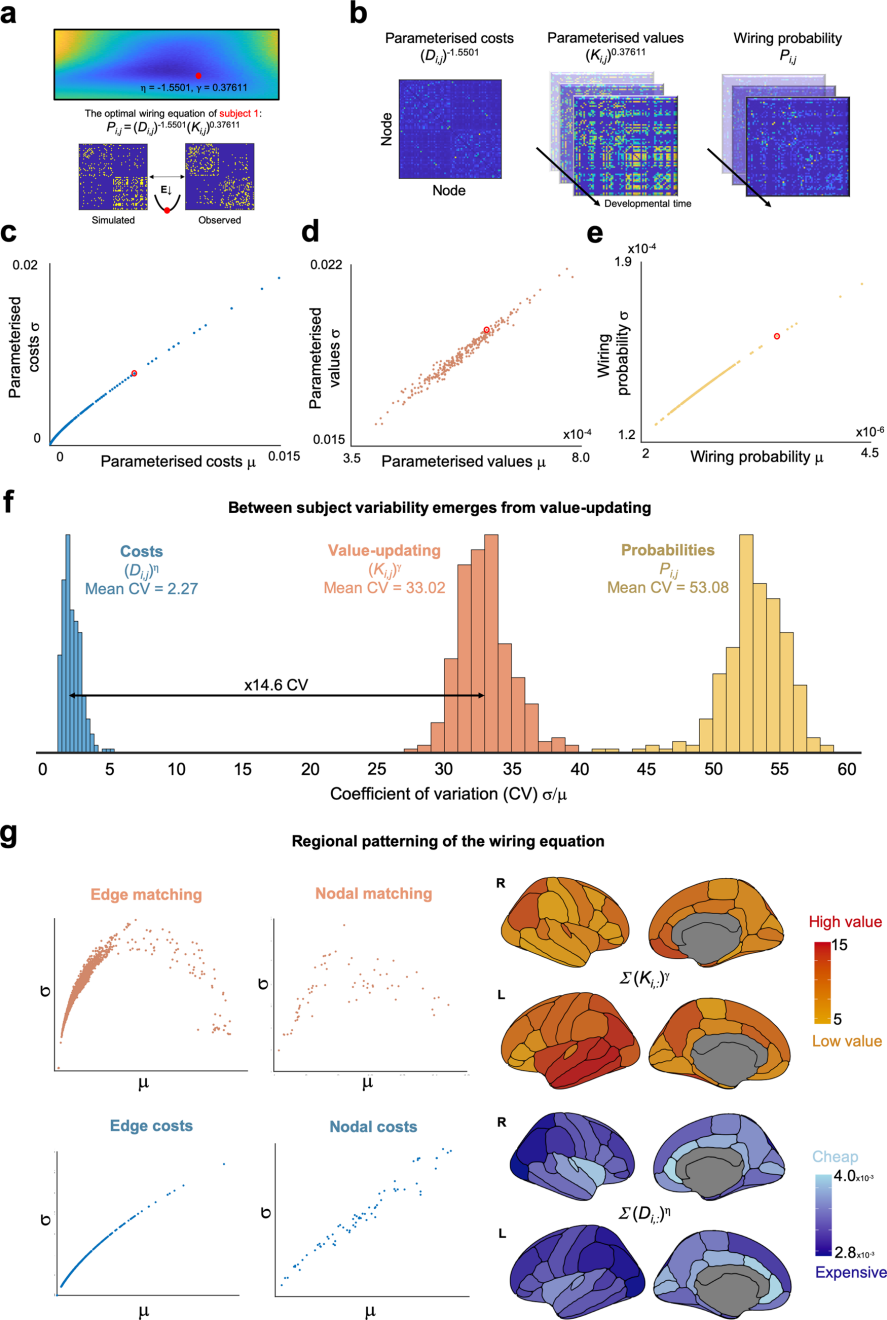
Wiring Eq. (1) decomposition and the subsequent variability across subjects in our heterogeneous sample. **a** For each subject, a simulated network is produced by minimizing the energy between the observed and simulated network. Here, we present visualizations for subject one (red). **b** Costs (*D*_*i,j*_) are static, while values (*K*_*i,j*_) dynamically update according to the matching rule, which enables the computation of wiring probability (*P*_*i,j*_). **c** The mean and standard deviation for each subject of their edge-wise parameterized costs, **d** parameterized values and **e** wiring probabilities. **f** Histograms of each subject’s coefficient of variation (CV) showing that subjects are more variable in their value-updating compared to costs, leading to large wiring probability variability. **g** Regional patterning of sample-averaged nodal parameterized costs and values, showing highly “valuable” patterning in the left temporal lobe and “cheap” regions generally occupying medial aspects of the cortex. Variability declines as value increases, but increases for costs.

While small generative parameter differences result in differential network properties, we have yet to show how this variability may occur over the development of the networks. That is, how do differences in parameter combinations across subjects manifest themselves when the network is developing? To address this, we examined how between-subject variability in optimal GNMs emerge at the level of cortical nodes and their connections. This is possible by simply decomposing the optimal simulation into its constituent parametrized costs (*D*_*i,j*_)^*η*^, values (*K*_*i,j*_)^*γ*^, and wiring probabilities (*P*_*i,j*_) at each time point, for each subject (Fig. 6a, b). This allows us to quantify growth trajectories and thus establish which aspects of network emergence vary most in the sample.

For each subject, we computed the coefficient of variation (CV, *σ*/*μ*) of their parameterized costs, matching values and wiring probabilities to compare subject-specific variability, as it emerges throughout the simulated growth of connectomes. While subjects exhibit some variability in how parameterized costs influence wiring probabilities (mean CV 2.27), this is dwarfed by their parameterized values over time (mean CV 33.02). This is because the matching value is dynamic, changing at each iteration (as in Fig. 1d), unlike relative Euclidean distance between nodes which is static. The result is that significant inter-individual variability arises in the probability of connections forming (mean CV 53.08), leading to the emergence of divergent brain organization (Fig. 6c–f). Furthermore, the regional patterning of costs and values is not random (Fig. 6g). Nodes and edges with high matching values decline in their variability, suggesting a consistency across subjects in highly “attractive” nodal structures and their connections. Across the sample, cheaper regions occupy the medial aspects of the cortex while highly valuable regions generally reside in the temporal cortex.

### Genomic patterning of network growth

Underlying these macroscopic changes in brain organization across time are a series of complex molecular mechanisms. These are partly governed by genetically coded processes that vary across individuals. We next tested whether these processes may steer the brain network toward a particular growth trajectory within our GNMs.

Nodal cost and nodal “matching” value patterning alongside regional gene expression profiles of 10,027 genes using human adult brain microarray data^41,42^ were integrated into two PLS analyses for each subject. For all analyses, gene expression scores at each node were used as the predictor. For each subject’s first analysis, their parameterized nodal costs (calculated as the subject’s *∑* (*D*_*i*_,:)^η^, as visualized Fig. 6g; fourth row, right) was used as the response variable. For each subject’s second analysis, their mean parameterized values (calculated as the subject’s (*∑* (*K*_*i*_,:)^*γ*^ averaged over time, as visualized in Fig. 6g; second row, right) was used as the response variable. Each analysis defined PLS components independently which were linear combinations of the weighted gene expression scores at each node (predictor variables) that were most strongly correlated with the subject’s nodal costs and nodal values of their simulated growth trajectory. To limit the variability across regions in terms of the samples available, only left hemispheric gene data were analyzed^42^.

**Fig. 7:**
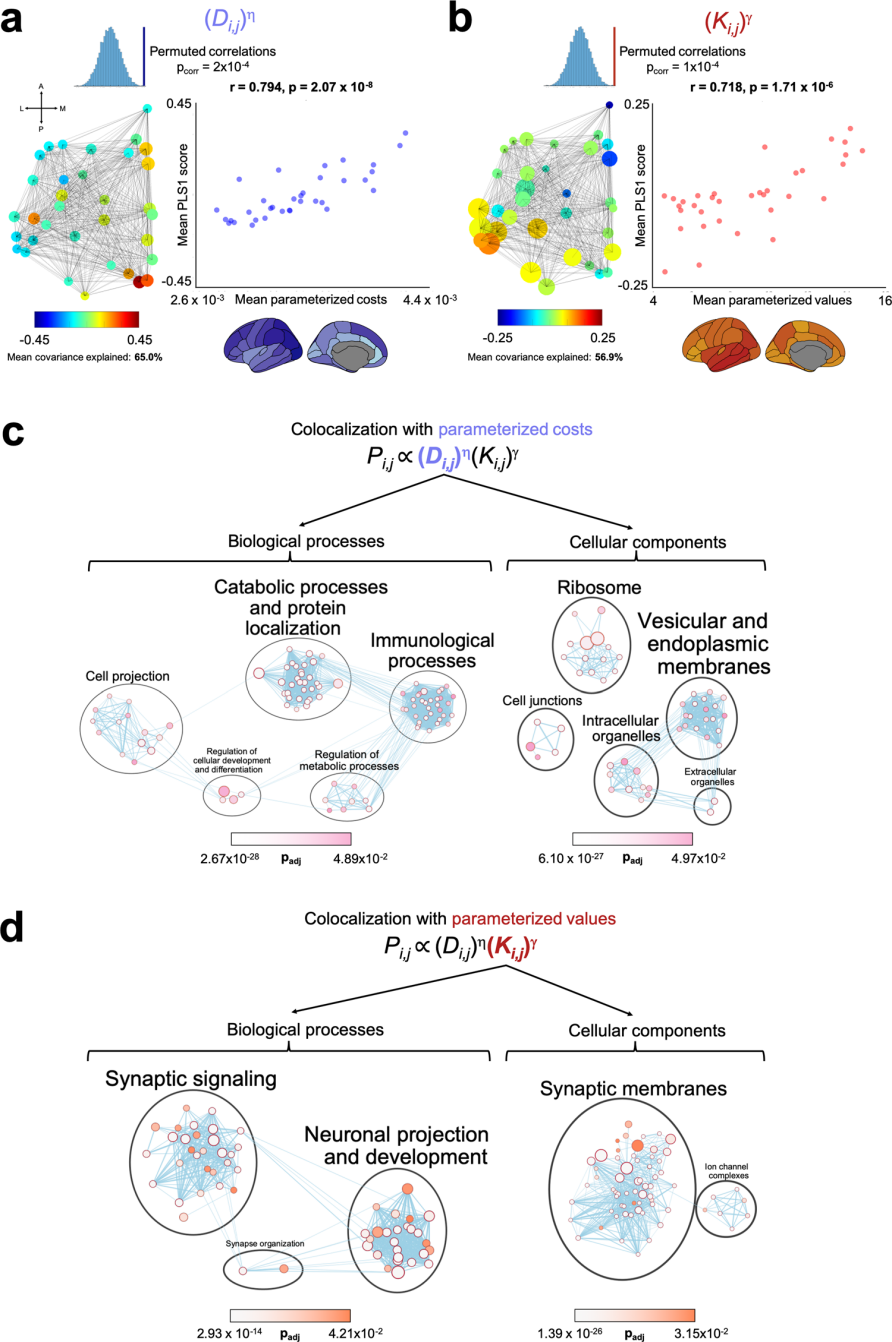
Over expressed genes which explain variance in brain wiring across subjects. Both PLS1 components across subjects are enriched for functionally specific biological processes and cellular components. Node size represents the number of genes in the set. The edges relate to gene overlap. **a** Sample-averaged parameterized costs significantly correlates with sample-averaged PLS1 nodal gene scores, explaining on average 65.0% covariance. Statistics were computed via two-tailed linear correlations, quoting the Pearson’s correlation coefficient, followed by *n* = 10,000 permutations. **b** Sample-averaged parameterized values significantly correlates with sample-averaged PLS1 nodal gene scores explaining on average 56.9% covariance. Statistics were computed via two-tailed linear correlations, quoting the Pearson’s correlation coefficient, followed by *n* = 10,000 permutations. **c** Nodal costs PLS1 is enriched for genes predominantly associated with protein localization, catabolic processes, and ribosomal/membrane cellular components. **d** Nodal values PLS1 is enriched for genes predominantly associated with synaptic signaling, neuronal projection and synaptic membranes.

Across our sample, the first PLS component (PLS1) explained on average 65.0% (SD 1.3%) and 56.9% (SD 9.2%) of the covariance between genetic expression and nodal costs, and nodal values, respectively. The average nodal costs PLS1 score significantly correlates with average nodal costs (*r* = 0.794, *p* = 2.07 × 10^−8^, *p*_corr_ = 2 × 10^−4^). Similarly, the average nodal values PLS1 score significantly correlates with average nodal values (*r* = 0.718, *p* = 1.71 × 10^−6^, *p*_corr_ = 1 × 10^−4^) (Fig. 7a, b). To then characterize the genetic profiles associated with each PLS analysis, we permuted the response variable 1000 times to form a null distribution for the loading of each gene, across each subject’s PLS1. This provides an estimate of how strong the loading would be by chance, and thus which genes exceed *p*_corr_ < 0.05. Across subjects, PLS1 provided an average of 581.5 significant genes (SD 101.4) for nodal costs and 437.6 significant genes (SD 167.4) for nodal values (Supplementary Fig. 8a).

Genes do not act in isolation, but instead converge to govern biological pathways across spatial scales. To move from individual genes to biological processes (BPs) and cellular components (CCs), we performed a pathway enrichment analysis^43^. Pathway enrichment analysis summarizes large gene sets as a smaller list of more easily interpretable pathways that can be visualized to identify main biological themes. Genes were ordered according to their frequency in being significantly associated with connectome growth across subjects for that component. For example, for nodal values PLS1, top of the list was the gene associated with connectome growth in the most subjects (*CHI3L1*; significant for 49.4% of our sample), the next was the second most frequent gene (*PRKAB2*; 36.4% of our sample) and so on. Our list stopped when genes were significant for <10% of the sample. This left the nodal costs PLS1 with a list of 1427 genes and the nodal values PLS1 with a list of 1584 genes ordered in terms of importance, which were submitted to pathway enrichment analysis in g:Profiler (https://biit.cs.ut.ee/gprofiler/gost) (Supplementary Fig. 8b)^43^. g:Profiler searches a collection of gene sets representing GO terms. In the ordered test, it repeats a modified Fisher’s exact test on incrementally larger sub-lists of the input genes and reports the sub-list with the strongest enrichment. Multiple-test correction is applied to produce an adjusted *p* value (*p*_adj_) for each enrichment^43,44^ (as visualized in Supplementary Fig. 8c, d, which can be accessed via the links presented in Supplementary Table 4).

The genes identified within the subject-wise PLS are not random, but instead converge on particular BPs and CCs. The nodal costs PLS1 was most prominently enriched for genes associated with BPs including catabolic processes and protein localization (32 BPs; all *p*_adj_ < 9.58 × 10^−3^), cell projection (14 BPs; all *p*_adj_ < 4.39 × 10^−2^), immunological processes (34 BPs; all *p*_adj_ < 4.82 × 10^−2^), regulation of metabolic processes (8 BPs; all *p*_adj_ < 4.75 × 10^−2^), and regulation of cell development and differentiation (4 BPs; all *p*_adj_ < 3.87 × 10^−2^). In terms of CCs, nodal costs PLS1 was enriched for genes associated with the ribosome (14 CCs; all *p*_adj_ < 2.15 × 10^−2^), vesicular and endoplasmic membranes (19 CCs; all *p*_adj_ < 4.90 × 10^−2^) and intracellular organelles (8 CCs; all *p*_adj_ < 4.97 × 10^−2^) (Fig. 7c).

The nodal values PLS1 was most prominently enriched for genes associated with BPs including synaptic signaling (29 BPs; all *p*_adj_ < 3.96 × 10^−2^), neuronal projection and development (26 BPs; all *p*_adj_ < 4.21 × 10^−2^), and synapse organization (2 BPs; all *p*_adj_ < 2.92 × 10^−2^). In terms of CCs, nodal values PLS1 was enriched for genes associated with synaptic membranes (60 CCs; all *p*_adj_ < 3.15 × 10^−2^) and ion channel complexes (7 CCs; all *p*_adj_ < 1.18 × 10^−2^) (Fig. 7d).

In Supplementary Table 4 we provide links so that readers can run our precise gene ontology (GO) queries within a browser and in Supplementary Fig. 8c, d we show a visualization of these enriched gene sets.

## Discussion

Diversity in macroscopic human brain organization can be modeled using a generative network. The generative framework does not include time itself as an explicit parameter, but instead models it as a sequence of processes, optimizing its connectivity by renegotiating its costs and value^24,25^ continuously over iterations. Despite the simplicity of this equation, it results in the dynamic updating of wiring probabilities over time, with multiple network properties, like spatial embedding, being an emergent property of this dynamic updating. This resonates with theoretical perspectives that implicate dynamic interactions between brain systems over development in progressive, integrative, specialization^45^. We have formalized this process in the context of neurodevelopmental diversity; offering a new perspective on the formation of organized macroscopic networks, their possible biological underpinnings, and their association with functional outcomes like cognitive performance. This reflects a theoretical step-change in understanding diversity in neurodevelopment, being sufficiently well-specified to generate macroscopic brain networks. In turn, this formalization allows for the unpacking of the computational and/or biological constraints that shape the trajectories of networks. Indeed, we anticipate that GNMs may be a powerful tool to model real and biologically feasible artificial networks across many scales.

Small changes in wiring parameters of the GNM lead to divergent macroscopic brain networks, with systematically different network properties. Within the model, the key factor that drives individual differences in growth trajectory is the dynamic nature of updating preferences over time. Specifically, as nodes form new connections this dynamically changes their neighborhoods, and in turn this quickly changes which nodes become “attractive” for subsequent connections. Importantly, individual differences in this process correspond significantly to independent structural data of the same individuals.

Why do the homophily-based generative rules approximate whole-brain networks so well? We propose that the superordinate goal of any developing brain network is to achieve the optimal computational capacity required of it, given finite biological resources. In this light, we suggest that matching produces the lowest-energetic networks precisely because it provides the closest heuristic estimate (compared to those tested here and in other works^24,25,36^) of the genuine dynamic reappraisals that occur over developmental time.

This is because by virtue of preferentially wiring with nodes with shared neighborhoods modular architectures emerge^46^, and this reflects the brain’s overarching structure. The modular architecture of the brain has been well studied, and has numerous properties enabling effective flexible computations likely important for functional integration^47^. By virtue of only requiring knowledge of neighborhood overlap, homophily-based methods may incur less informational costs^48^ relative to other methods which require global information, and therefore may be more biologically plausible. It is of note that homophily is a measure somewhat akin to network communicability—another locally knowable measure containing information that closely relates to the shortest path^49^. Finally, a tentative explanation for homophily at the neuronal level can also be provided in terms of Hebbian-like plasticity^24,50^ This opens up the exciting possibility for these generative models to model developing neuronal cultures^51^ to explore whether similar wiring principles may operate across scales.

Our current GNMs operate at a whole-brain level—i.e., a global set of rules governing network formation. But with more biologically realistic information about regional differences it is possible that an alternative growth model could be fitted^52^. Unlike a generative model, a growth model captures the graded changes of an established network over time. This would allow for time itself to be incorporated as a parameter within the model, making regionally and temporally sensitive modifications to network growth and therefore encompass multiple longitudinal measurements of the same individual over a biologically meaningful timescale. The only work we know of to have done this is by Nicosia et al.^52^ in which data from Caenorhabditis elegans (with birth times of ~300 neurons) where integrated into a growth model to reproduce the developing C. elegans network and it is bi-phasic growth rate. With sufficient detailed information it may be possible in future to take a similar growth modeling approach in humans. Regional variation in nodal costs and values closely mirrored the expression profiles for different sets of genes, which in turn govern different BPs and CCs. Since the advent of genome-wide association studies (GWAS), a huge number of genes have been implicated in developmental disorders, including schizophrenia^53^ and autism^54^, but also general cognitive functioning^55^. It has been challenging to interpret the consequences of these individual implicated genes. The enrichment analysis that accompanied our GNM takes a very different approach. As far as we are aware, this is the first study aiming to bridge models of whole brain organizational emergence and genetics in this way (for work utilizing various generative models, see^24,25,36,50,56^ and for work that integrates Allen Human Brain Atlas gene data with functional and structural brain imaging, see^57–61^). Nodal costs covaried with genes enriched for highly costly metabolic processes, including catabolic processes, protein transport and CCs centered around the ribosome and endoplasmic membranes. On the other hand, nodal values covaried with genes enriched for trans-synaptic signaling, neuronal projection and the synaptic membrane. This aligns with recent findings that synaptic genes also colocalize with highly synergistic regions of the brain, which have been suggested to be crucial for human cognitive evolution^61^.

The omnigenic model^62^ suggests that complex traits are driven by genes that do not have direct effects on the trait per se, but instead propagate through regulatory networks on much smaller numbers of core genes, with more direct effects. This model explains the vast number of GWAS hits for complex traits, as “peripheral” genes necessarily outnumber “core” genes and thus the sum of their small effects exceeds the contribution of core genes. We suggest the omnigenic model may apply to some aspects of gene-development relationships. That is, the many genes that contribute to each PLS1 may not directly contribute to developmental processes themselves, but in the regulation of activity and growth within brain areas that are particularly important for neurodevelopment. Crucially there is variability in enriched genes across subjects (Supplementary Fig. 8a).

Our sample is a large mixed cohort of children, the majority of whom were referred from specialists in children’s educational and clinical services. This was the ideal testbed for exploring diverse trajectories. The varied referral routes for the cohort makes its composition more reflective of children at heightened neurodevelopmental risk, relative to a more standard case-control design recruited according to strict diagnostic inclusion and exclusion criteria, via a single referral route^63^. But it is important to note that the modeling also works well in a more typical sample recruited from classrooms in the same area. So, whilst the CALM cohort is ideal for exploring the mechanisms of heterogeneity, these same mechanisms are likely at play in more typical samples. Indeed, this work presents a challenge to the long history of categorizing neurodevelopment into discrete groupings based on observed cognitive and/or behavioral traits. Instead, we suggest divergent outcomes may arise via slight trajectory changes that fall out of the continual negotiation of brain connectivity optimization. While likely that generative preferences are initialized via an individual’s genetic preprograming, small changes in wiring preferences over time—possibly via complex interactions of their time course, endocrinological exposure, learning and environment—have profound effects on the emergence of the developmental trajectory. What results is a continual interaction between network growth preferences and the dynamically developing brain, leading to neurodiverse outcomes.

Whilst our sample is designed to capture children at risk, the findings generalized to a more typical sample, with an even split of boys and girls, recruited in local schools. This suggests that this computational approach could be a powerful tool for developmental scientists more generally. The advent of larger datasets is allowing the study of the developing brain at unprecedented scale and across multiple levels^64,65^. Computational frameworks that allow the integration of different datatypes (e.g., multiple imaging modalities, genetic variability) could provide a valuable tool for building developmental theory that goes beyond correlating different datatypes over time, and fully capitalizes on the scale and complexity of those datasets. In the future, this approach could be used to test different theoretical accounts of developmental change, and to make longitudinal predictions where multiple waves of data are available. To realize these opportunities, a crucial next step is to deploy this type of computational modeling to capture the diversity present in larger population-level neuroimaging datasets, with longitudinal data^64–66^ spanning multiple sites.

This computational framework has a number of limitations that provide scope for future improvements. Our generative models are limited to the binary connections which are assumed to be anatomical. This is inevitably a gross simplification of the complex weighted structure of the connectome. Devising ways in which network connections can change in a more graded fashion is a necessary next step to modeling more complete developmental processes. In the future we will need to capture both the strengthening and weakening of connections that has been shown to occur in human brain development^67,68^. Secondly, we currently use one rule, but it is conceivable that different rules govern growth at different points in the trajectory. It may be possible to accurately approximate the rules governing the remodeling of networks over time, modeled either by changing heuristic estimates (e.g., changing of generative rules over time) or attempting to optimize a superordinate goal (e.g., computational efficiency and/or flexibility). Thirdly, our gene enrichment results are correlational, not causative. There remains an explanatory gap in determining whether and how these specific gene profiles support the sensitivity to connection formation. And crucially, the expression data are derived for a microarray analysis of postmortem tissue samples from human adults^41,42^. Moreover, while RNA-seq data were used to cross-validate gene expression measures by removing probes with a correlation <0.2, it was not possible to externally validate the data with an independent dataset. Caution is therefore required when interpreting our enrichment analysis due to this lack of external validation^69^. The next steps will involve validating these findings in large-scale developmental cohorts with available gene data, and forming casual links by applying GNMs to individuals with neurodevelopmental disorders of known genetic origin^58,59,70^. Fourthly, we have used a parcellation widely used in developmental studies^71–75^, which aids the comparability of our findings with the wider literature. Previous work has shown that parcellation choice is not a big determent of optimal wiring parameters^25^. But within the context of a developmental sample there could be subtle differences in the optimal parcellation across participants, and thus the production of individually optimized parcellations^76,77^ would allow us to test whether and how developmental change in the parcellation itself influence wiring properties. Finally, as in previous studies^24,25,36^, our models utilize Euclidean distance as measures of cost in connection formation. While a simplification, this selection removes any a priori constraints to the generative model that a more biologically specified cost measure may provide (e.g., fiber lengths, which are sparse and thus limit potential connections). A number of studies have shown relatively inconsistent findings in terms of how much Euclidean distance accounts for fiber length, with findings ranging from 22% (RED, in this work) to 79% (Human Connectome Project (HCP)) of variance explained. There may be cohort, parcellation, and tractography effects influencing these relationships. Whilst interpolated fiber lengths have been shown to perform equivalently to Euclidean distances^25^ within GNMs, it is important to consider that in our modeling these two measures only partially overlap. A crucial next step is to test whether microstructural informed tractography^78^, which may provide a more direct measure of biological cost, improves model performance.

In conclusion, we provide a unifying computational framework for conceptualizing the emergence of structural brain networks and their diversity. The emergence of brain networks can be understood as occurring via continual renegotiations of costs and values, but individuality emergences from their slightly different parameterization.

## Methods

**Fig. 8:**
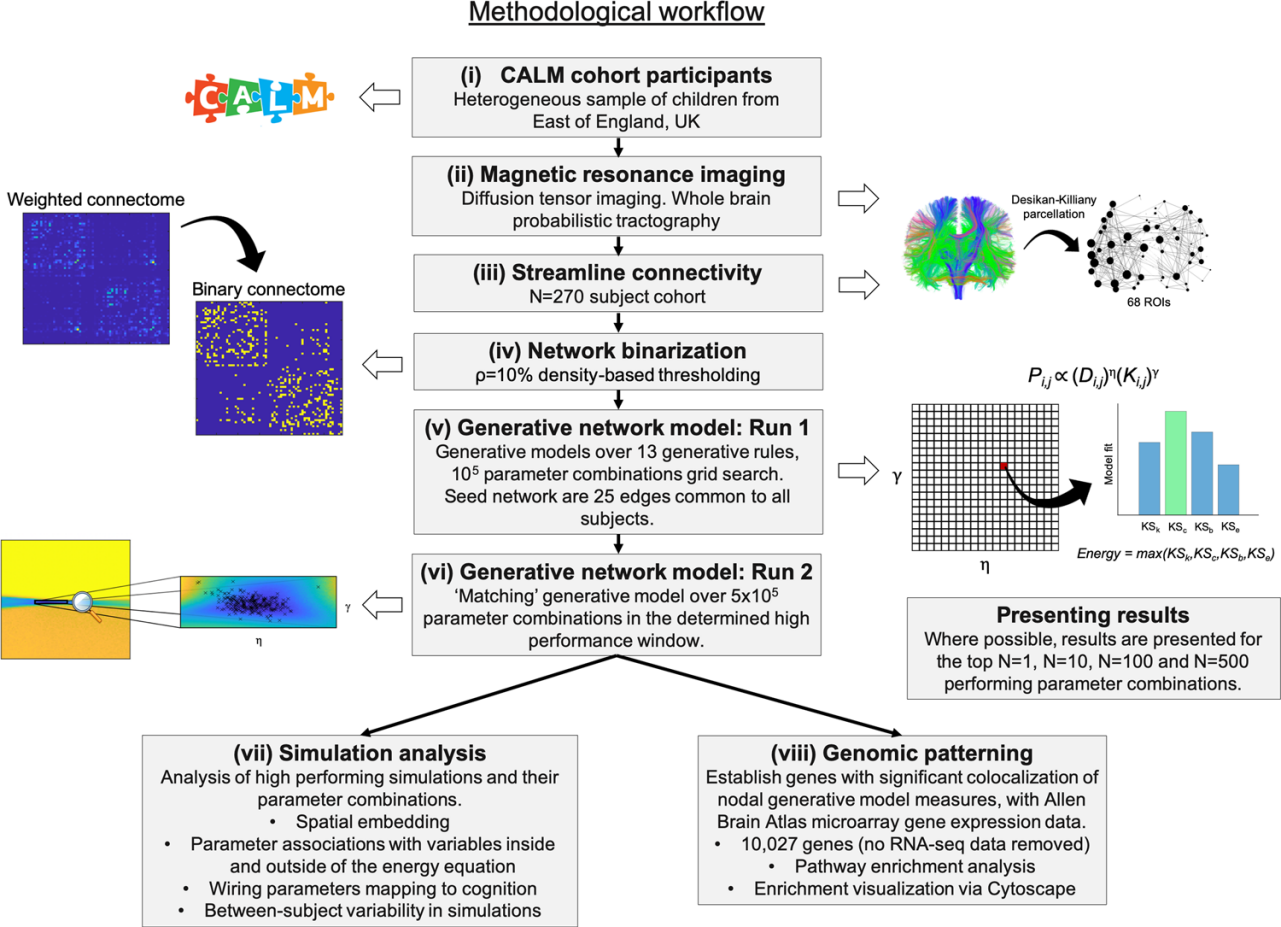
Schematic of the methodological workflow. The basic workflow involved (i) Recruitment of the CALM cohort, a heterogeneous referred sample from the East of England (UK) with wide inclusion criteria; (ii) MRI diffusion tensor imaging; (iii) Estimation of structural connectivity within the Desikan–Killiany parcellation; (iv) Binarization of the connectome; (v) Initial run of the GNM for all 13 generative rules as outlined in Supplementary Table 1; (vi) More specific run of the homophily “matching” GNM in the narrow parameter window; (vii) Further analysis of simulations in terms of spatial embedding, parameter associations and variability; (viii) Combination of Allen Human Brain Atlas data and the generative model findings.

The methodological workflow is summarized in Fig. 8.

### Participants

The sample were made up of children referred by practitioners working in specialist educational or clinical services in the East of England (UK) to the Centre for Attention Learning and Memory (CALM), a research clinic at the MRC Cognition and Brain Sciences Unit, University of Cambridge (see Holmes et al.^35^ for the full protocol of assessment, and refs. ^9–13^ for prior work using the same cohort). The composition of this cohort is design to be broadly reflective of children at heightened neurodevelopmental risk for poor developmental outcomes. Consent was obtained from parents and assent was obtained from all youngsters. The study protocol was approved by, and data collection proceeded under the permission of, the local NHS Research Ethics Committee (reference: 13/EE/0157). This cohort of children is intentionally heterogenous. Referrers were asked to identify children with cognitive problems related to learning, with primary referral reasons including difficulties with ongoing problems in “language”, “attention”, “memory”, or “learning/poor school progress”. Exclusion criteria were uncorrected problems in vision or hearing, English as a second language, or a causative genetic diagnosis. Children could have single, multiple, or no formally diagnosed learning difficulty or neurodevelopmental disorder. Most referrals were made from Special Educational Needs Coordinators (57.0%), followed by Pediatricians (24.1%) and Speech and Language Therapists (4.2%) (Supplementary Fig. 9a, b). Subsequently the CALM team supplemented the cohort with a smaller set of unreferred children, recruited from the same schools and neighborhoods, so that the cohort captures the full ability spectrum. The CALM cohort contains *n* = 967 total children (*N* = 805 referred; *N* = 162 unreferred). Of these, *N* = 299 undertook MRI scanning of which *N* = 279 had usable MRI data (see “MRI acquisition and preprocessing”). *N* = 270 of these had cognitive data available (see “Cognitive and learning assessments”) (see Supplementary Fig. 9c for a visualization of the cognitive variability across the cohort). This sample includes 65.9% boys, mean age 117.8 months, age range was 66–223 months and 78 that came from the non-referred comparison sample. The increased ratio of boys to girls is what we would expect from epidemiology studies of children at neurodevelopmental risk of poor learning or clinical outcomes^79^.

To validate our modeling, we also included a second cohort of *n* = 140 typically developing children, who had been recruited from local schools (the RED cohort; mean age 9.34 years, SD age 1.41 years, range 6.82–12.8 years, 45.7% boys). These data were collected under the permission of the Cambridge Psychology Research Ethics Committee (references: Pre.2013.34; Pre.2015.11; Pre.2018.53). Parents/legal guardians provided written informed consent and all children provided verbal assent. This second dataset was previously reported by Johnson et al.^37^. In Supplementary Table 5, we provide all demographic information of the cohorts used in the study. Race and ethnicity data for the CALM and RED cohorts are not yet available, but are to be published^35^.

### MRI acquisition and preprocessing

MRI data were acquired at the MRC Cognition and Brain Sciences Unit in Cambridge, on the Siemens 3 T Prisma-fit system (Siemens Healthcare) using a 32‐channel quadrature head coil. T1‐weighted volume scans were acquired using a whole brain coverage 3D Magnetization Prepared Rapid Acquisition Gradient Echo sequence acquired using 1 mm isometric image resolution. Echo time was 2.98 ms, and repetition time was 2250 ms. Diffusion scans were acquired using echo‐planar diffusion‐weighted images with an isotropic set of 68 noncollinear directions, using a weighting factor of *b* = 1000 s mm^−2^, interleaved with 4 T2‐weighted (*b* = 0) volume. Whole brain coverage was obtained with 60 contiguous axial slices and isometric image resolution of 2 mm. Echo time was 90 ms and repetition time was 8500 ms. Both CALM and RED samples underwent the same scanning protocol. *N* = 299 CALM and *N* = 167 RED children underwent MRI scanning. Twenty (CALM) and 27 (RED) scans were not useable due to excessive motion (>3 mm movement during the diffusion sequence estimated through FSL eddy), leaving an MRI sample of *N* = 279 CALM and *N* = 140 RED children, respectively.

### Connectome construction and cortical morphology

MRI scans were converted from the native DICOM to compressed NIfTI‐1 format. Next, correction for motion, eddy currents, and field inhomogeneities was applied using FSL eddy. Furthermore, we submitted the images to nonlocal means de‐noising^80^ using DiPy v0.11^81^ to boost signal‐to‐noise ratio. A constant single angle model was fitted to the 60‐gradient‐direction diffusion‐weighted images using a maximum harmonic order of 8 using DiPy. Whole‐brain probabilistic tractography was performed with 8 seeds on all voxels. The step size was set to 0.5 and the maximum number of crossing fibers per voxel to 2. For ROI definition, T1‐weighted images were submitted to nonlocal means denoising in DiPy, robust brain extraction using ANTs v1.9^82^, and reconstruction in FreeSurfer v5.3 (http://surfer.nmr.mgh.harvard.edu). Regions of interest (ROIs) were based on the Desikan–Killiany parcellation of the MNI template^83^ with 34 cortical ROIs per hemisphere. FreeSurfer v5.3 was used for tissue classification and anatomical labeling. The technical details of these procedures are described elsewhere^84–86^. FreeSurfer morphology statistics were computed for each ROI.

To construct the connectivity matrix, the number of streamlines intersecting both ROIs was estimated and transformed into a density map for each pairwise combination of ROIs. A symmetric intersection was used so that streamlines starting and ending in each ROI were averaged. Self-connections were removed. To produce binarized connectomes from the resulting 68-by-68 streamline matrix, we enforced an average connectome density of *ρ* = 10% (as in Betzel et al.^25^), resulting in a streamline threshold of 27 streamlines (i.e., a minimum of 27 streamlines must have connected two regions for us to consider the presence of an anatomical connection).

### Generative network models

Starting with a sparse seed network (25 bi-directional edges that were common across all *N* = 270 subjects), edges were added one at a time over a series of steps until a total number of connections were placed that equaled that of the target observed connectome (group level connections, mean = 231.4 and SD = 19.1) (As shown in Supplementary Fig. 1d). The same process was separately done for the validation cohort. Each step allows for the possibility that any pair of unconnected nodes will be connected. Connections are formed probabilistically, where the relative probability of connection formation, between nodes *i and j*, is given by Eq. (1). We used 13 previously studied non-geometric rules^24,25^ to produce energy landscapes. *D*_*i,j*_ was defined as the Euclidean distance between node centroids. (*D*_*i,j*_)^*η*^ was computed as a power-law, as shown previously to have better performance than exponentials^24^. Euclidean distance accounts for 24 and 22% of the variance in fiber length in the CALM and RED samples respectively. These estimates are relatively low by comparison with other cohorts, with previous studies showing Euclidean distance to account for 32% (Nathan Kline Institute, Rockland, New York), 66% (CHUV; University Hospital Center and University of Lausanne), and 79% (HCP) of the variance in fiber length^25^. Topological parameters were computed using our own internally developed functions adapted from the Brain Connectivity Toolbox (https://sites.google.com/site/bctnet/)^87^.

To evaluate the fitness of synthetic networks and optimize models, we defined an energy function that measures how dissimilar a synthetic network is to the observed network as defined by Betzel et al.^25^. This is given in Eq. (2). Initially, we ran simulations across a defined a parameter space of 10,000 evenly spaced combinations (−7 ≤ *η* ≤ 7, −7 ≤ *γ* ≤ 7), for each generative rule (Fig. 2a–d). Parameter ranges were based on approximate values that had been evaluated in previous work^24,25,36^. This was to capture their respective energy landscapes and to estimate their relative effectiveness at generating plausible networks. We then computed a further a set of 50,000 simulations within a much narrower low-energy window (−3.606 ≤ *η* ≤ 0.354 and 0.212 ≤ *γ* ≤ 0.495) of the matching algorithm (Fig. 2f) for all subsequent analysis. This is because the matching algorithm attained the lowest-energy networks and therefore best approximated individual-level connectomes. In Supplementary Fig. 2, we computed the SD of the top *n* = 500 performing (lowest energy) wiring parameters to determine how variable the solutions are.

### Cognitive and learning assessments

A large battery of cognitive, learning, and behavioral measures was administered in the CALM clinic^35^. *N* = 9 CALM children did not have available cognitive data (of the *N* = 279 MRI sample) and were therefore excluded, leaving the final sample *N* = 270 children. These children had no missing data. All cognitive scores were age-standardized, controlling for age. For full details of the processing of cognitive data, see Siugzdaite et al.^13^.

The following nine measures of fluid and crystallized reasoning were included: Matrix Reasoning, a measure of fluid intelligence^88^ (Wechsler Abbreviated Scale of Intelligence); Peabody Picture Vocabulary Test^89^. Phonological processing was assessed using the Alliteration subtest of the Phonological Awareness Battery^90^. Verbal and visuo‐spatial short‐term and working memory were measured using Digit Recall, Dot Matrix, Backward Digit Recall, and Mr X subtests from the Automated Working Memory Assessment^91,92^. Learning measures (literacy and numeracy) were taken from the Wechsler Individual Achievement Test II^93^ and the Wechsler Objective Numerical Dimensions^94^, apart from 78 of controls for which we used multiple subtests from the Woodcock Johnson for Verbal ability^95^.

### Gene expression data

Regional microarray expression data were obtained from six postmortem brains provided by the Allen Human Brain Atlas (http://human.brain-map.org/)^41,42^ which, as far as we are aware, is the only publicly available human 3D brain map of the transcriptome which covers the full cortex. Of note, other datasets such as the BrainSpan Atlas for the Developing Human Brain (http://brainspan.org) are available, although currently covering 16 ROI.

The Allen Human Brain Atlas dataset is based on microarray analysis of postmortem tissue samples from six human donors aged between 18 and 68 years with no known history of neuropsychiatric or neurological conditions. Data were imported from Arnatkevičiūtė et al.^42^. Since only two of the six brains included samples from the right hemisphere, analyses were conducted on the left hemisphere only. Probes where expression measures do not exceed the background in >50% samples were removed and genes that did not have a corresponding RNA-seq measure were removed. To improve validity of the gene dataset by cross-validating expression measures, probes with a Spearman’s correlation <0.2 with external RNA-seq data were removed, and a representative probe with the highest correlation to RNA-seq data was selected for each gene. Sample assignment was computed by applying a 2 mm distance threshold. In total, a mean of 37.8 ± 22.5 (SD) samples were assigned to each ROI (min = 5; max = 92)^42^.

The fully preprocessed gene data comprised of a 34 by 10,027 matrix of microarray array gene expression data. These data were used for a subsequent PLS analysis (see “Statistics”; “PLS analysis”) and pathway enrichment analysis (see “Gene enrichment analysis and visualization”).

### Statistics

#### Predictions of spatial embedding

To assess the performance of the optimal matching GNMs to produce networks with spatial embedding of topological characteristics, we averaged across each subject’s best performing simulation (which achieved the lowest energy; descriptive statistics shown in the top row of Supplementary Table 2) to produce a single 68 (ROIs) by one vector for each measure. We did the same for their observed connectomes. Figure 3 shows their linear correlations. In Supplementary Fig. 4a, we run the same process for local efficiency (not included in the energy equation). For Supplementary Fig. 4b–e, we correlate the same networks, but not for spatial embedding as these are global network measures outside of the energy equation. Subsequently, we determined the generative error (i.e., mismatch) for the simulations in terms of each statistical network property within the energy equation at the node-level. This was done as a simple subtraction of the observed from the simulated network (as shown in Supplementary Fig. 5). The absolute mean ranked error was then calculated by taking the modulus of the generative error, averaging across the four statistical properties, and taking the rank order. All network measures were calculated using functions from the Brain Connectivity Toolbox^87^.

#### Global associations of parameters with graphical and morphological measures

In Fig. 4 and Supplementary Table 3, we present group level correlational analysis between *η*, *γ* and observed global graph theory and cortical morphology measures. In each case, the observed connectome and morphological measures were averaged across the whole cortex.

#### PLS analysis

We used PLS regression to address two distinct aspects of the study. First, we used PLS to determine the latent components of the wiring equation and connectome features which best explain cognitive task performance (Fig. 5a). The *p*_cov_ and *p*_corr_ significance values of each component were determined by permuting the cognitive data 10,000 times and comparing the observed covariance (*p*_cov_) and coefficient of determinations (*p*_corr_) relative to their null distributions. Figure 5b, c shows the correlation of predictor and response scores and response and predictor loadings of the significant PLS1 component (*p*_cov_ = 0.009 and *p*_cov_ = 0.049 in terms of covariance explained and *p*_corr_ = 7 × 10^−4^ and *p*_corr_ = 4 × 10^−4^ in terms of correlation coefficient) for each analysis, respectively. Second, we used PLS to identify the linear combinations of genes that best predicted average nodal costs and values each subject’s optimal simulation (as outlined previously). For this analysis, subject 162 was removed from as they were the only subject to have an optimally performing *γ* that was positive, which biased results due to being an outlier (as parametrized values are calculated as (*K*_*i,j*_)^*γ*^), leaving a sample of *N* = 269. For each of the *N* = 269 subjects, two PLS analyses were performed, providing 538 separate PLS analyses. We performed permutations of the correlations between average scores and costs/values in the same way as previously to determine significance of the PLS modeling across the sample. To assess the significance of each gene in terms of its loading, we ran *N* = 1000 permutations of the response variable for each PLS. This allowed us to compute a gene loading *p*_corr_ for each component of the PLS which was collapsed across subjects (as visualized in Supplementary Fig. 8b) for gene enrichment analysis (see “Gene enrichment analysis and visualization”).

#### Variability in the decomposed wiring equation

To determine where variability arises in the growth of the networks, we decomposed the wiring equation for each subject. This was achieved by first running the optimal wiring equation for each subject and taking their cost (a static Euclidean distance matrix), matching and wiring probability matrices at each step in the network growth model. For each subject, we took all edges that existed within the simulation and computed their mean and SD (Fig. 6c–e) and then determined their CV (Fig. 6f shows their distributions). To then explore within-connectome variability, we performed the same analysis but collapsing across subjects to determine how nodes (summed rows of the matrix) and edges (elements of the matrix) vary (Fig. 6g).

### Gene enrichment analysis and visualization

We next aimed to elucidate the BPs and CCs for which our gene lists converged on. A BP is defined as representing a specific objective that the organism is genetically programmed to achieve. A BP is accomplished by a particular set of molecular functions carried out by specific gene products (or macromolecular complexes), often in a highly regulated manner and in a particular temporal sequence (https://www.ebi.ac.uk/QuickGO/term/GO:0008150) On the other hand, a CC is defined as a location, relative to cellular compartments and structures, occupied by a macromolecular machine when it carries out a molecular function. There are two ways in which the GO describes locations of gene products: (1) relative to cellular structures (e.g., cytoplasmic side of plasma membrane) or compartments (e.g., mitochondrion), and (2) the stable macromolecular complexes of which they are parts (e.g., the ribosome) (https://www.ebi.ac.uk/QuickGO/term/GO:0005575).

To elucidate BPs and CCs across the sample, genes with a *p*_corr_ < 0.05 following permutation testing on each component were deemed significant. This provided an individual-level vector of genes that were significant for an individual for each of the nodal costs and nodal values PLS1. To collapse across subjects, genes were then ordered according to their frequency in being significantly associated with connectome growth across subjects for that component. The list stopped when genes were significant for <10% of the sample. For each subject, PLS1 provided an average of 581.5 significant genes (SD 101.4) for nodal costs and 437.6 significant genes (SD 167.4) for nodal values (Supplementary Fig. 8a). When collapsed across subjects as described, the nodal costs PLS1 had 1427 genes and the nodal values PLS1 had 1584 genes ordered in terms of importance, which were then submitted to a pathway enrichment analysis.

For all information as to the enrichment and visualization pipeline, please refer to Reimand et al.^43^. In short, GO annotations are the most commonly used resource for pathway enrichment analysis. g:Profiler^44^ (https://biit.cs.ut.ee/gprofiler/gost) searches a collection of gene sets representing GO terms and, in the ordered test, repeats a modified Fisher’s exact test on incrementally larger sub-lists of the input genes and reports the sub-list with the strongest enrichment. Multiple-test correction is applied to produce an adjusted *p* value (*p*_adj_)^43,44^ (as visualized in Supplementary Fig. 8c, d, which can be accessed via the links presented in Supplementary Table 4). To visualize enriched pathways, we used “EnrichmentMap” within Cytoscape v3.8.0 (http://www.cytoscape.org)^43,96^. All default parameters were used. Pathways are shown as nodes (representing enriched BPs) that are connected by edges if the pathways share genes. Nodes are colored by their *p*_adj_ and edges are sized on the basis of the number of genes shared by the connected pathways. To then identify clusters of themes, AutoAnnotate v1.3.3 was used before manually curating the suggested theme names to accurately reflect all pathways within each theme.

### Reporting summary

Further information on research design is available in the Nature Research Reporting Summary linked to this article.

## Supplementary information

Supplementary Information

Reporting Summary

## Data Availability

The datasets supporting the current study have not been deposited in a public repository because of restrictions imposed by NHS ethical approval, but are available from the corresponding author on request. Requests for access can be made by research-based institutions for academic purposes. A response can be expected within 1 week. Unidentifiable simulated data can be found at https://osf.io/h9px4/?view_only=984260dcff444b59819961ece9c724ec.

## Code availability

Results were generated using code written in R, Python and MATLAB. All code is available at https://github.com/DanAkarca/generativenetworkmodel^97^.
