# Supplementary material for: A generative network model of neurodevelopmental diversity in structural brain organization
Source: Nat Commun. 2021 Jul 9;12:4216. doi: 10.1038/s41467-021-24430-z (PMC8270998; doi:10.1038/s41467-021-24430-z)
Supplement: Supplementary file 1 — Supplementary Information [file 41467_2021_24430_MOESM1_ESM.pdf]

Supplementary Information:

A generative network model of neurodevelopmental diversity in structural brain organization

Danyal Akarca[1*], Petra E Vértes[2,3], Edward T Bullmore[2,4], the CALM team[1], & Duncan E Astle[1]

[1]MRC Cognition and Brain Sciences Unit, University of Cambridge, Cambridge, UK
[2]Department of Psychiatry, University of Cambridge, Cambridge, UK
[3]The Alan Turing Institute, London, UK
[4]Department of Clinical Neurosciences, Wolfson Brain Imaging Centre, University of Cambridge, Cambridge, UK

*danyal.akarca@mrc-cbu.cam.ac.uk

Supplementary Figures

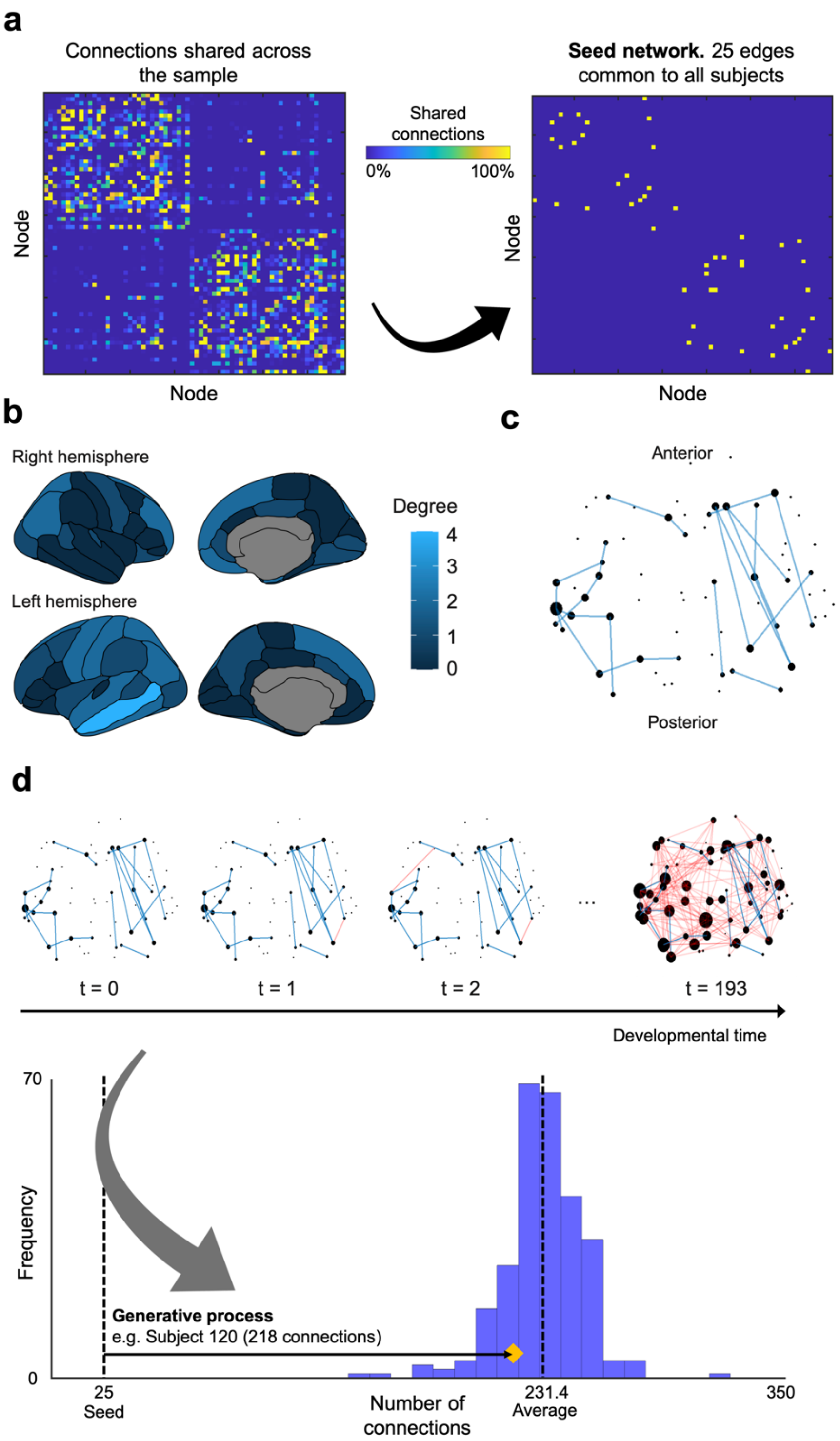

Supplementary Fig. 1 Seed network construction and generative model overview. a The seed network was constructed by taking connections that were shared across 100% of the sample, as in Betzel, R.F. *et al*[25]. This left 25 bidirectional connections that were common across all subjects. **b** The degree distribution of the seed network **c** An edge-wise view of the seed network. Node size relates to degree. **d** A schematic illustration of how generative models aim to uncover the wiring rules that give rise to an observed brain network. Starting from the seed network (10.8% density of the average observed network), edges are added to the network according to parameterized costs and value (i.e. rule) terms until it reaches the same number of edges as observed. For this illustration, subject 120 is shown, where 193 edges are added to the seed's 25 edges, leaving 218 edges in the final network. The distribution of number of observed edges are shown, with a mean of 231.4 edges and standard deviation of 19.1.

Rule			Energy		η		γ	
Name	Class	$K_{i,j}$	Mean	SD	Mean	SD	Mean	SD
Spatial	Spatial	*1*	0.337	0.020	-3.940	0.397	n/a	n/a
Neighbours	Homophily	$\sum_w a_{iw} a_{wj}$	0.165	0.020	-1.995	0.483	0.338	0.045
Matching	Homophily	$\frac{\lvert \Gamma i \backslash j \cap \Gamma j \backslash i \rvert}{\lvert \Gamma i \backslash j \cup \Gamma j \backslash i \rvert}$	0.141	0.021	-1.647	0.490	0.357	0.022
C-Average	Cluster	$\frac{Ci}{2} + \frac{Cj}{2}$	0.181	0.016	-3.070	0.378	-6.203	0.735
C-Minimum	Cluster	$min(C_i, C_j)$	0.252	0.020	-3.626	0.396	-6.131	0.795
C-Maximum	Cluster	$max(C_i, C_j)$	0.182	0.014	-3.095	0.387	-6.153	0.735
C-Difference	Cluster	$\lvert C_i - C_j \rvert$	0.322	0.025	-6.193	0.744	-3.480	2.012
C-Product	Cluster	$C_i C_j$	0.223	0.024	-3.401	0.393	-5.964	0.926
D-Average	Degree	$\frac{ki}{2} + \frac{kj}{2}$	0.221	0.019	-3.516	0.502	2.419	0.332
D-Minimum	Degree	$min(k_i, k_j)$	0.279	0.016	-4.749	0.749	0.474	0.010
D-Maximum	Degree	$max(k_i, k_j)$	0.216	0.019	-3.557	0.602	2.255	0.332
D-Difference	Degree	$\lvert k_i - k_j \rvert$	0.229	0.018	-3.836	0.669	1.689	0.577
D-Product	Degree	$k_i k_j$	0.277	0.018	-4.605	0.738	0.399	0.082

Supplementary Table 1 Generative rules, their formulae and descriptive statistics across the broad parameter space. Descriptive statistics of the η and γ parameters required to achieve the best performing network for each subject, across each of the generative rules over the space defined ($-7 \leq \eta \leq 7$, $-7 \leq \gamma \leq 7$) for 10,000 simulations.

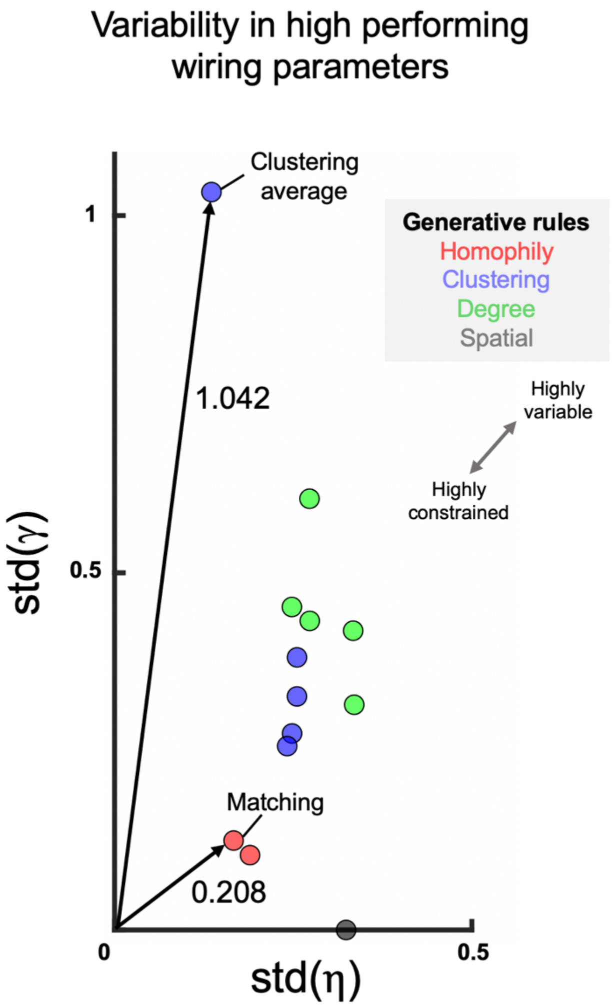

Supplementary Fig. 2 Wiring parameter variability across the n=500 lowest energy simulations. Each point corresponds to a coordinate denoting the standard deviation of the n=500 lowest energy η (x-axis) and γ (y-axis) parameters for each generative rule. Two vectors are plotted for the rule with the lowest variability (Matching) with a Euclidean of 0.208, and the highest variability (Clustering average) with a Euclidean of 1.042.

Matching: Narrow energy window						
Averaged over top N networks	Energy		η		γ	
	Mean	SD	Mean	SD	Mean	SD
N = 1	0.090	0.010	-1.565	0.327	0.349	0.033
N = 10	0.101	0.011	-1.579	0.334	0.352	0.036
N = 100	0.116	0.013	-1.614	0.372	0.355	0.038
N = 500	0.1326	0.015	-1.658	0.427	0.357	0.040

Supplementary Table 2 Descriptive statistics of high performing wiring parameters η and γ, and the energy of the networks they produce. Parameters were selected using the matching generative rule with 50,000 simulations, evenly spaced across the narrow window $-3.606 \leq \eta \leq 0.354$ and $0.212 \leq \gamma \leq 0.495$. Parameters were averaged across a variable N number of high performing wiring combinations to determine stability.

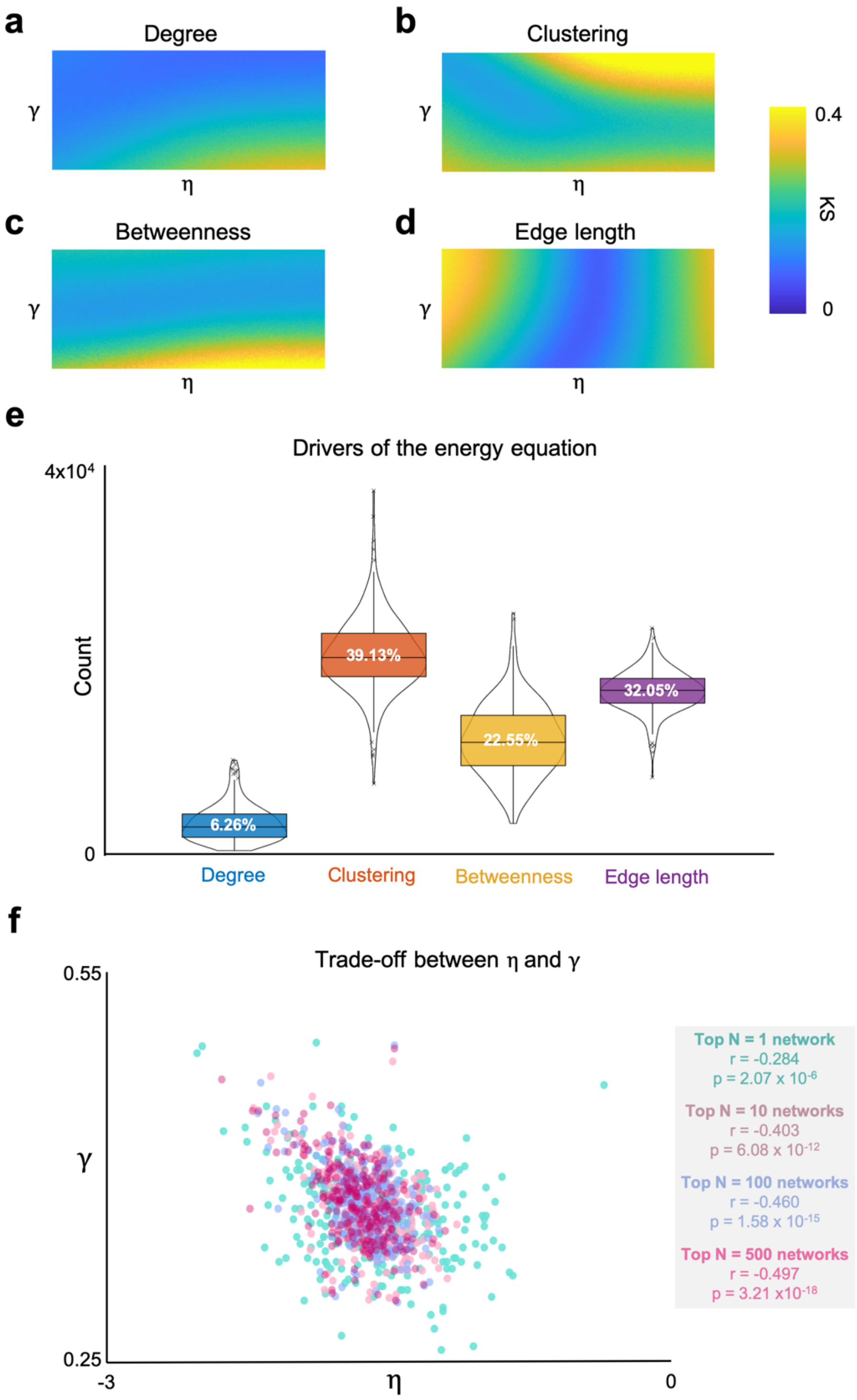

Supplementary Fig. 3 Exploration of energy and KS statistics, and the resultant parameter combination relationships. a Visualisation of group-averaged degree KS statistics in the window **b** Clustering coefficient KS statistics **c** Betweenness-centrality KS statistics **d** Edge length statistics. **e** The KS statistics which were "maxed" as to produce the energy statistic, across all subjects and simulations in the narrow parameter window. In total, there are N = 270 x 50,000 = 13,500,000 simulations. On average, the energy equation was driven by clustering 39.13% (N = 5,282,550) of the time, followed by edge length at 32.05% (N = 4,326,750), followed by betweenness at 22.55% (N = 3,044,250) and degree at 6.26% (N = 845,100). The boxplot presents the median and IQR. Outliers are demarcated as small black crosses, and are those which exceed 1.5 times the interquartile range away from the top or bottom of the box. **f** Within the narrow window, η and γ trade-off such that subjects with a larger γ have a greater preponderance to have a larger magnitude negative η. All statistics were computed via two-tailed linear correlations, quoting the Pearson's correlation coefficient.

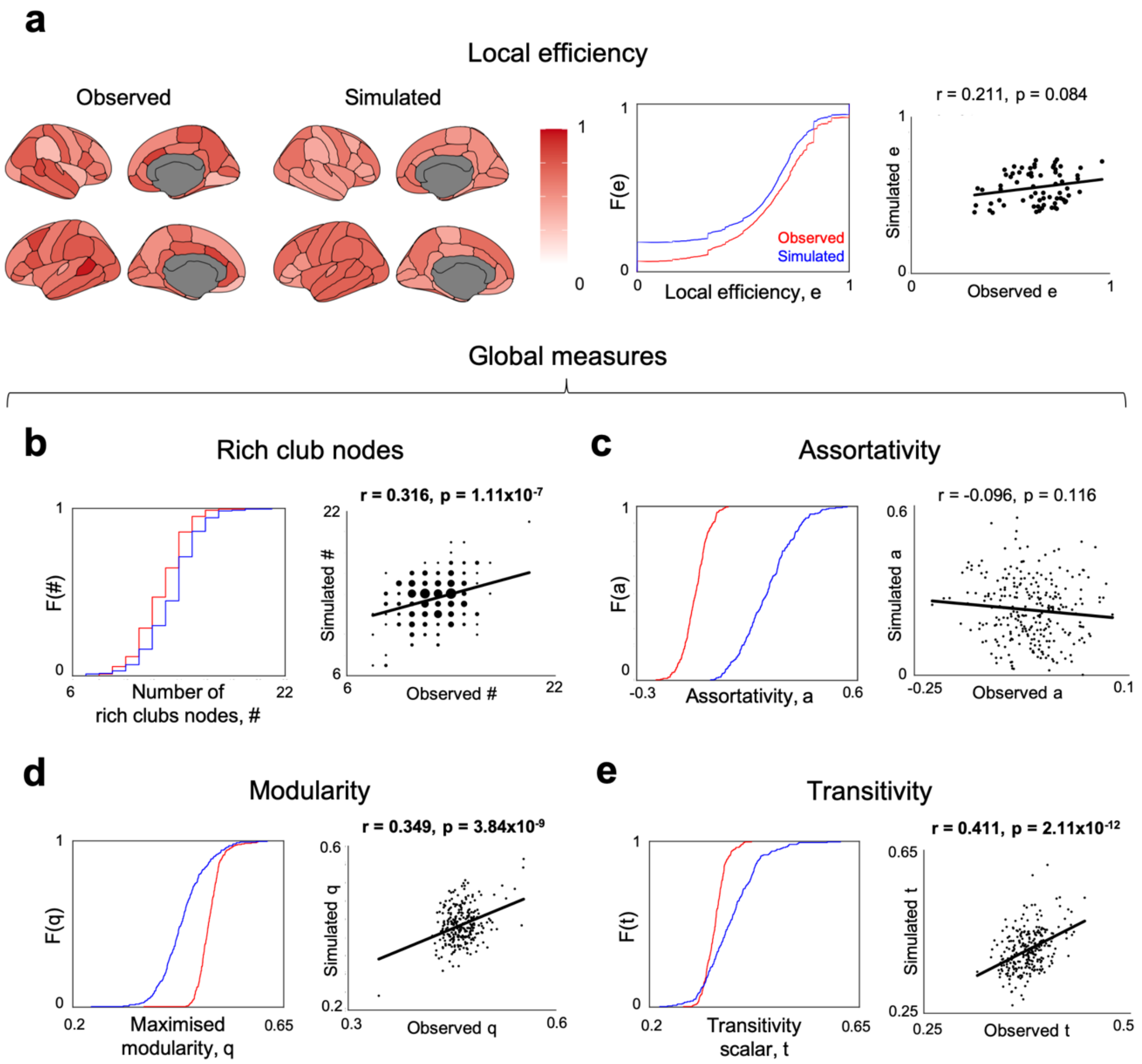

Supplementary Fig. 4 Out-of-sample network measures, not included in the energy equation, and their relationships between simulated and observed measures. Local efficiency is the only local measure, and thus, as in Figure 3, each point in the scatter plot reflects the across-subject average nodal measure from observed and optimal simulated networks. All other measures are global and therefore each point in the scatter represents a subject in the sample. All statistics were computed via two-tailed linear correlations, quoting the Pearson's correlation coefficient. (**a**) Local efficiency between observed and simulated networks are not significantly correlated ($r = 0.211$, $p = 0.084$). (**b**) The number of rich club nodes between observed and simulated networks are significantly positively correlated ($r = 0.316$, $p = 1.11 \times 10^{-7}$) (**c**) Assortativity between observed and simulated networks are not significantly correlated ($r = -0.096$, $p = 0.116$) (**d**) Modularity between observed and simulated networks are significantly positively correlated ($r = 0.349$, $p = 3.84 \times 10^{-9}$) (**e**) Transitivity between observed and simulated networks are significantly positively correlated ($r = 0.411$, $p = 2.11 \times 10^{-12}$). Boldened values are significant correlations at $p < 0.05$.

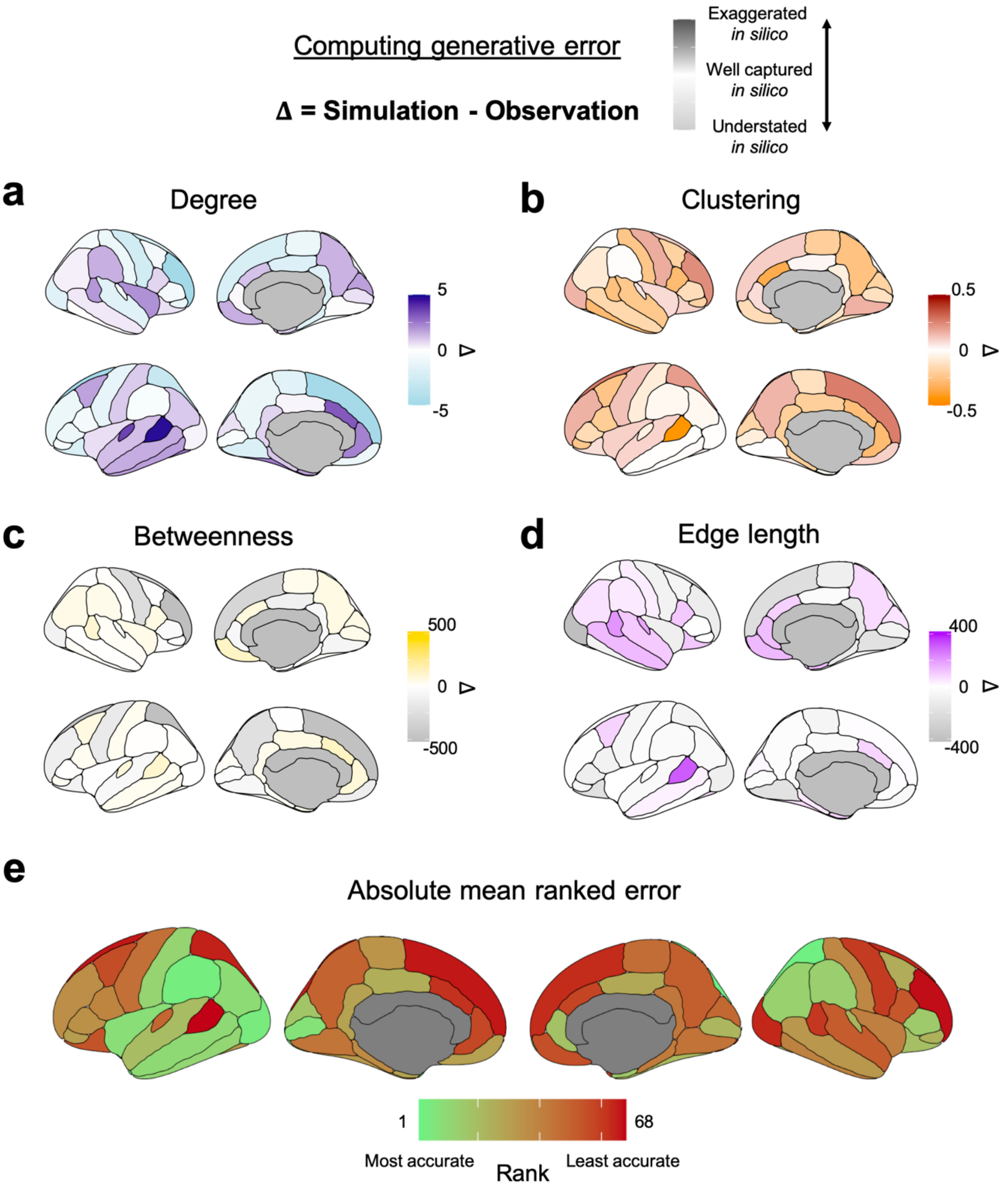

Supplementary Fig. 5 Computing the generative mismatch between low energy simulated networks and those observed. a. Degree generative errors. **b** Clustering generative errors **c** Betweenness centrality generative errors. **d** Edge length generative errors. **e** Visualisation of the absolute mean ranked error across the four measures, indicating the accuracy to which regions are approximated via the optimised homophily generative models.

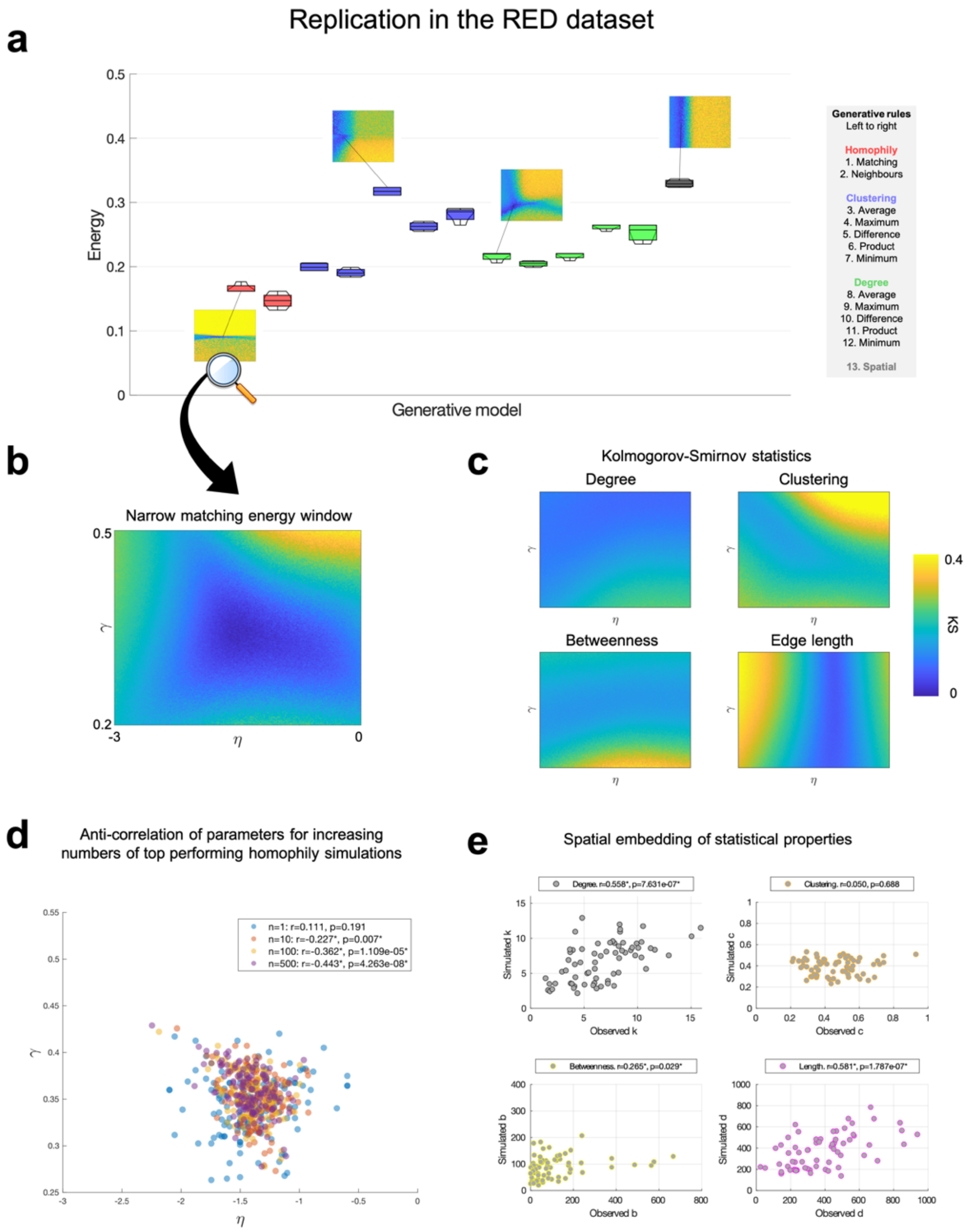

Supplementary Fig. 6 Replication of key findings in the external n=140 RED dataset. For details regarding the cohort, see *Methods; cohort characteristics*. **a** Initial generative models were run in the same way as CALM, across thirteen generative rules via an evenly spaced grid-search for 10,000 simulations ($-7 \leq \eta \leq 7$, $-7 \leq \gamma \leq 7$) per rule (130,000 simulations in total). Connectomes were averaged across the sample and thresholded via the same procedure, to produce a single representative connectome on which the generative models were fit. Boxplots represent the highest n=5 performing simulations for this representative connectome. The boxplot presents the median and IQR. Four broad parameter spaces are shown to highlight how they mirror landscapes as shown in Figure 2. **b** The homophily energy window was then computed in the same way as before, with n=50,000 simulations being run for each subject. This visualisation shows the energy window after being averaged over each subject's window. **c** The corresponding Kolmogorov-Smirnov statistics mirror those shown in CALM in Supplementary Fig. 3a-d. **d** We find a very similar pattern of parameter anti-correlations in the homophily window, and that negative correlations increase with increasing numbers of high performing parameters (n=1, n=10, n=100 and n=500) as shown in Supplementary Fig. 3f. Statistics were computed via two-tailed linear correlations, quoting the Pearson's correlation coefficient. **e** We replicate key findings that spatial embedding occurs as a result of the generative homophily model, as shown in Fig. 3. All statistics were computed via two-tailed linear correlations, quoting the Pearson's correlation coefficient.

Measure	η								γ							
	Top N=1 simulated network		Top N=10 simulated networks		Top N=100 simulated networks		Top N=500 simulated networks		Top N=1 simulated network		Top N=10 simulated networks		Top N=100 simulated networks		Top N=500 simulated networks	
	r	p	r	p	r	p	r	p	r	p	r	p	r	p	r	p
Age	**0.1370**	**0.0244**	**0.2473**	**3.9791x10^{-5}**	**0.3194**	**8.0719x10^{-8}**	**0.3253**	**4.5182x10^{-8}**	**-0.1479**	**0.0150**	**-0.1451**	**0.0170**	**-0.1217**	**0.0458**	-0.1174	0.0541
Total streamlines	-0.1080	0.0763	**-0.2740**	**4.9125x10^{-6}**	**-0.3852**	**5.5782x10^{-11}**	**-0.4251**	**2.8187x10^{-13}**	**0.4309**	**1.2449x10^{-13}**	**0.5652**	**3.4216x10^{-24}**	**0.5649**	**3.6420x10^{-24}**	**0.5634**	**5.1759x10^{-24}**
Degree	0.0623	0.3074	-0.0828	0.1748	**-0.1209**	**0.0472**	**-0.1845**	**0.0023**	**0.3215**	**6.5867x10^{-8}**	**0.5765**	**2.5715x10^{-25}**	**0.5860**	**2.7400x10^{-26}**	**0.5947**	**3.2074x10^{-27}**
Clustering coefficient	**-0.2424**	**5.7241x10^{-5}**	**-0.4312**	**1.1915x10^{-13}**	**-0.5754**	**3.3105x10^{-25}**	**-0.6338**	**9.7869x10^{-32}**	**0.5879**	**1.7144x10^{-26}**	**0.7878**	**2.3807x10^{-58}**	**0.8314**	**2.4001x10^{-70}**	**0.8285**	**1.8709x10^{-69}**
Betweenness centrality	**-0.1595**	**0.0087**	-0.1128	0.0641	**-0.1741**	**0.0041**	**-0.1391**	**0.0223**	**-0.2194**	**2.8060x10^{-4}**	**-0.4125**	**1.6248x10^{-12}**	**-0.4225**	**4.0817x10^{-13}**	**-0.4424**	**2.2968x10^{-14}**
Edge length	**0.3415**	**8.4813x10^{-9}**	**0.4752**	**1.2926x10^{-16}**	**0.5477**	**1.5660x10^{-22}**	**0.4925**	**6.6608x10^{-18}**	0.1168	0.0553	**0.2094**	**5.3257x10^{-14}**	**0.2287**	**1.5018x10^{-4}**	**0.2491**	**3.4712x10^{-5}**
Efficiency	0.1049	0.0854	0.041	0.5020	0.0154	0.8005	-0.0460	0.4516	**0.2070**	**6.2060x10^{-4}**	**0.3988**	**9.9888x10^{-12}**	**0.4158**	**1.0312x10^{-12}**	**0.4340**	**7.8898x10^{-14}**
Number of rich club nodes	0.0861	0.1581	0.0177	0.7718	0.0090	0.8827	0.0206	0.7360	**0.2041**	**7.4004x10^{-4}**	**0.3560**	**1.7377x10^{-9}**	**0.3832**	**7.1277x10^{-11}**	**0.4001**	**8.4373x10^{-12}**
Assortativity	0.0130	0.8313	0.0358	0.5578	0.0311	0.6104	0.0531	0.3845	**-0.1850**	**0.0023**	**-0.1964**	**0.0012**	**-0.1564**	**0.0101**	**-0.1602**	**0.0083**
Transitivity scalar	**-0.2548**	**2.2667x10^{-5}**	**-0.3989**	**9,7813x10^{-12}**	**-0.5281**	**8.5927x10^{-21}**	**-0.5767**	**2.4941x10^{-25}**	**0.4863**	**1.9646x10^{-17}**	**0.6383**	**2.6553x10^{-32}**	**0.6792**	**7.0291x10^{-38}**	**0.6689**	**2.1791x10^{-36}**
Maximised modularity	**-0.2249**	**1.9449x10^{-4}**	**-0.1747**	**0.0040**	**-0.2626**	**1.234x10^{-5}**	**-0.2374**	**8.1647x10^{-5}**	-0.1052	0.0844	**-0.2291**	**1.4583x10^{-4}**	**-0.2432**	**5.3864x10^{-5}**	**-0.2543**	**2.3398x10^{-5}**
Number of vertices	0.0061	0.9207	-0.0950	0.1196	**-0.1461**	**0.0163**	**-0.1627**	**0.0074**	**0.1329**	**0.0291**	**0.2214**	**2.4517x10^{-4}**	**0.2141**	**3.9639x10^{-4}**	**0.2081**	**5.7950x10^{-4}**
Surface area	-0.0040	0.9482	-0.1048	0.0856	**-0.1644**	**0.0068**	**-0.1799**	**0.0030**	**0.1492**	**0.0141**	**0.2363**	**8.8556x10^{-5}**	**0.2278**	**1.5917x10^{-4}**	**0.2217**	**2.4095x10^{-4}**
Grey volume	-0.0331	0.5885	-0.0909	0.1362	**-0.1730**	**0.0044**	**-0.1979**	**0.0011**	**0.1262**	**0.0382**	**0.2058**	**6.6834x10^{-4}**	**0.1938**	**0.0014**	**0.1871**	**0.0020**
Mean thickness	-0.0603	0.3236	0.0323	0.5976	-0.0100	0.8698	-0.0296	0.6283	0.0026	0.9661	-0.0025	0.9676	-0.0137	0.8222	-0.0146	0.7697
Std thickness	-0.1031	0.0909	**-0.1570**	**0.0098**	**-0.1682**	**0.0056**	**-0.1685**	**0.0055**	0.0828	0.1747	0.0137	0.8231	-0.0115	0.8508	-0.0146	0.8112
Mean curvature	-0.0435	0.4770	**-0.1378**	**0.0235**	**-0.1510**	**0.0130**	**-0.1259**	**0.0387**	0.0508	0.4061	0.0447	0.4642	0.0294	0.6306	0.0222	0.7167
Gaussian curvature	-0.0222	0.7170	-0.0560	0.3593	0.0035	0.9543	0.0012	0.9842	0.0344	0.5740	-0.0809	0.1848	-0.0780	0.2015	-0.0861	0.1582
Folding index	0.0396	0.5172	-0.0683	0.2635	**-0.1281**	**0.0354**	-0.1110	0.0686	0.0531	0.3852	0.0670	0.2727	0.0637	0.2974	0.0519	0.3954

Supplementary Table 3 Correlational statistics between η and γ and observed global network and graphical measures. Global measures were calculated by averaging across the cortex. Parameters were averaged across a variable N number of best performing networks to determine how this influences associations. Rows 3 – 6 (blue) correspond to measures minimised within the energy equation (2). Rows 7 – 11 (green) correspond to measures not included in the energy equation. Rows 12 – 19 (yellow) are cortical morphology measures which were not included in the energy equation. All statistics were computed via two-tailed linear correlations, quoting the Pearson's correlation coefficient. Boldened values represent associations which are significant at $p < 0.05$.

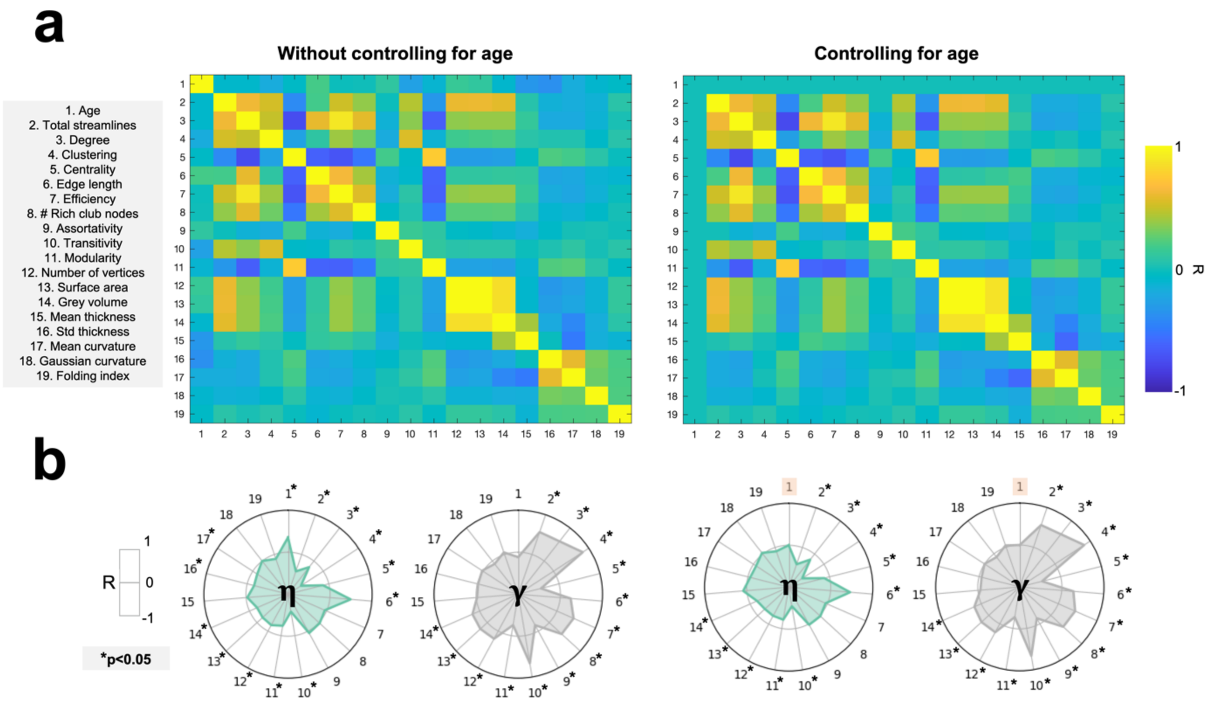

Supplementary Fig. 7 Controlling for age has little influence on the relationships between statistical measures, and how they relate to wiring parameters. a Correlation matrices between statistical measures without controlling for age (Left; as shown in Fig. 4a) and when age has been controlled (Right) appear near identical. **B** Radar plots of the correlation coefficients between η and γ and each statistical measure. The left two radar plots are the same as shown in Fig. 4b. The right two radar plots are equivalent but when controlling for age, reflected by the orange box over the first measure which represents age. All correlations remain significant apart from η with morphological measures 16. std. thickness and 17. mean curvature.

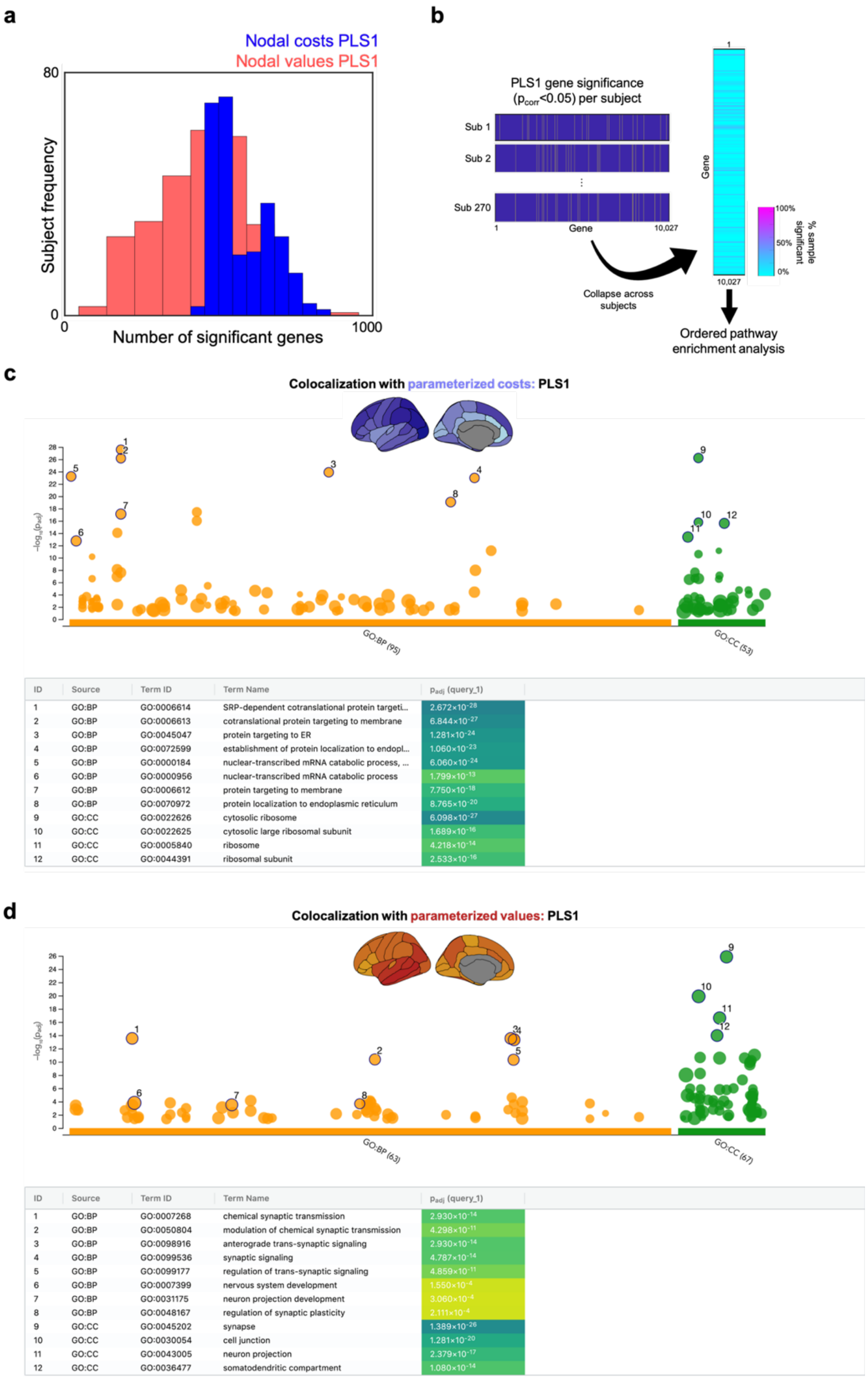

ID	Source	Term ID	Term Name	p_{adj} (query_1)
1	GO:BP	GO:0006614	SRP-dependent cotranslational protein targeti...	2.672×10^{-28}
2	GO:BP	GO:0006613	cotranslational protein targeting to membrane	6.844×10^{-27}
3	GO:BP	GO:0045047	protein targeting to ER	1.281×10^{-24}
4	GO:BP	GO:0072599	establishment of protein localization to endopl...	1.060×10^{-23}
5	GO:BP	GO:0000184	nuclear-transcribed mRNA catabolic process, ...	6.060×10^{-24}
6	GO:BP	GO:0000956	nuclear-transcribed mRNA catabolic process	1.799×10^{-13}
7	GO:BP	GO:0006612	protein targeting to membrane	7.750×10^{-18}
8	GO:BP	GO:0070972	protein localization to endoplasmic reticulum	8.765×10^{-20}
9	GO:CC	GO:0022626	cytosolic ribosome	6.098×10^{-27}
10	GO:CC	GO:0022625	cytosolic large ribosomal subunit	1.689×10^{-16}
11	GO:CC	GO:0005840	ribosome	4.218×10^{-14}
12	GO:CC	GO:0044391	ribosomal subunit	2.533×10^{-16}

ID	Source	Term ID	Term Name	p_{adj} (query_1)
1	GO:BP	GO:0007268	chemical synaptic transmission	2.930×10^{-14}
2	GO:BP	GO:0050804	modulation of chemical synaptic transmission	4.298×10^{-11}
3	GO:BP	GO:0098916	anterograde trans-synaptic signaling	2.930×10^{-14}
4	GO:BP	GO:0099536	synaptic signaling	4.787×10^{-14}
5	GO:BP	GO:0099177	regulation of trans-synaptic signaling	4.859×10^{-11}
6	GO:BP	GO:0007399	nervous system development	1.550×10^{-4}
7	GO:BP	GO:0031175	neuron projection development	3.060×10^{-4}
8	GO:BP	GO:0048167	regulation of synaptic plasticity	2.111×10^{-4}
9	GO:CC	GO:0045202	synapse	1.389×10^{-26}
10	GO:CC	GO:0030054	cell junction	1.281×10^{-20}
11	GO:CC	GO:0043005	neuron projection	2.379×10^{-17}
12	GO:CC	GO:0036477	somatodendritic compartment	1.080×10^{-14}

Supplementary Fig. 8 Gene loading analysis and pathway enrichment. a Distributions of significant genes (p_{corr} < 0.05) for the nodal-costs and nodal-values PLS1, which provided an average of 581.5 significant genes and 437.6 significant respectively across the sample. **b** To collapse across subjects, we ranked genes according to the number of subjects for which each gene was significant (according to n = 1000 permutations, p_{corr}<0.05) and submitted genes above 10% to an ordered pathway enrichment analysis, using g:Profiler. Pathway enrichment analysis summarises large gene sets as a smaller list of more easily interpretable pathways that can be visualised to identify main biological themes. **c** Parameterized costs pathway enrichment analysis, for biological processes and cellular components, and **d** Parameterized values.

PLS	Response	# Genes	Gene Ontology	Link
PLS1	Parametrised costs	1427	Biological processes and cellular components	https://biit.cs.ut.ee/gplink/l/TYGoJHihSs
	Parameterised values	1584	Biological processes and cellular components	https://biit.cs.ut.ee/gplink/l/H_gK8UIVR1

Supplementary Table 4 Gene queries submitted to g:Profiler. Ordered gene lists were computed by taking subject-specific significant (p_{corr} <0.05) genes according to their frequency across the sample, up to 10% of the sample.

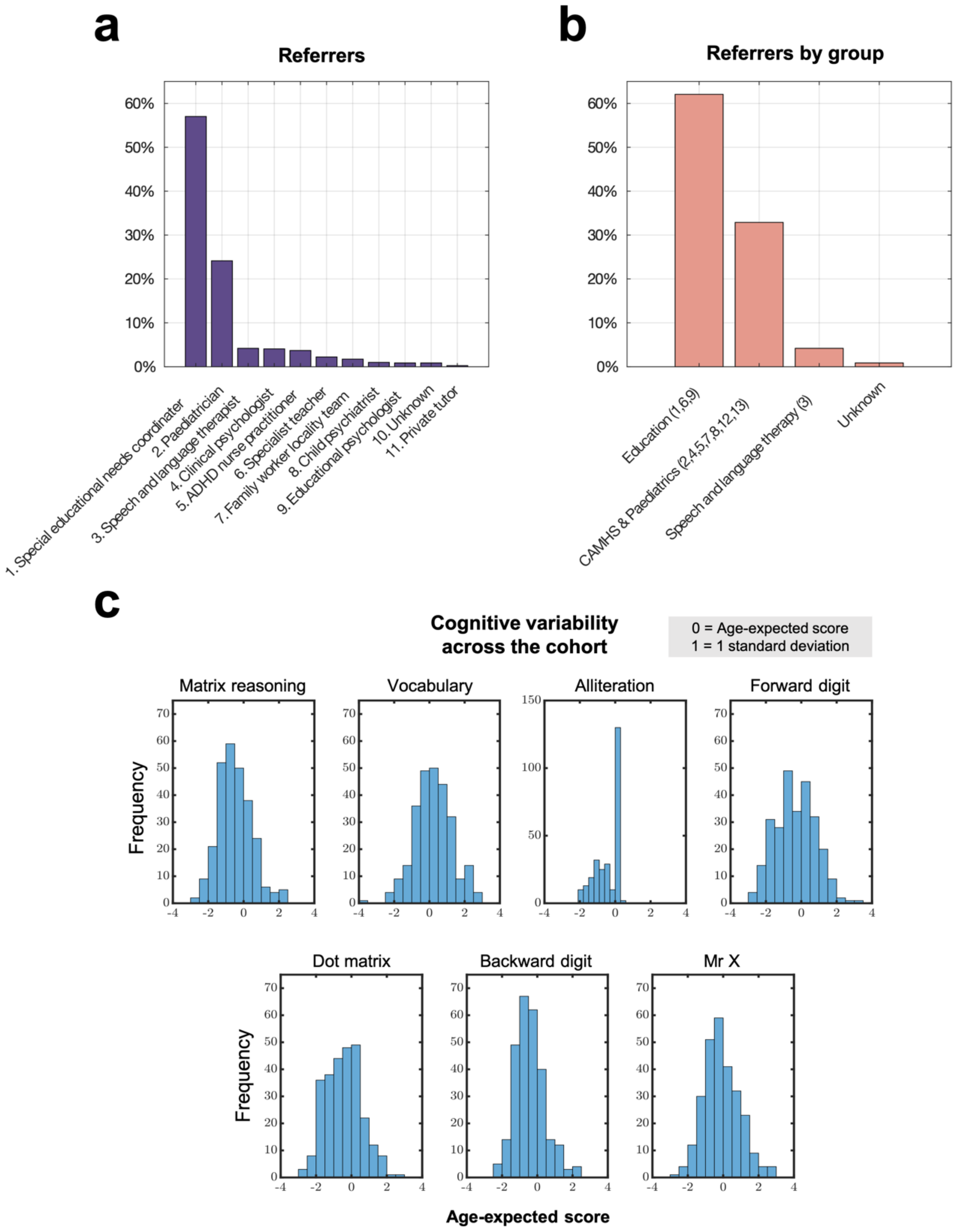

Supplementary Fig. 9 Referral rates and cognitive variability across the CALM cohort. a The majority of referrals came from Special Educational Needs Coordinators followed by Paediatricians. **b** Referrers by broad group. CAMHS; Child and Adolescent Mental Health Services. **C** Age-expected scores across the seven cognitive variables.

Cohort	CALM	RED
**n**	**270**	**140**
**Age (years)**		
Mean	9.82	9.34
SD	2.15	1.41
Range	5.50-18.60	6.82-12.8
**Sex**		
Male	178	64
Female	92	76
**Referred**		
Yes	192	n/a
No	78	n/a

Supplementary Table 5 Demographic information of cohorts used. Samples include the main CALM and validation RED cohorts.
