# Supplementary material for: A generative network model of neurodevelopmental diversity in structural brain organization
Source: Nat Commun. 2021 Jul 9;12:4216. doi: 10.1038/s41467-021-24430-z (PMC8270998; doi:10.1038/s41467-021-24430-z)
Supplement: Supplementary file 2 — Reporting Summary [file 41467_2021_24430_MOESM2_ESM.pdf]

nature research

Corresponding author(s): Dr Danyal Akarca

Last updated by author(s): May 21, 2021

Reporting Summary

Nature Research wishes to improve the reproducibility of the work that we publish. This form provides structure for consistency and transparency in reporting. For further information on Nature Research policies, see our Editorial Policies and the Editorial Policy Checklist.

Statistics

For all statistical analyses, confirm that the following items are present in the figure legend, table legend, main text, or Methods section.

n/a	Confirmed	
☐	☒	The exact sample size (n) for each experimental group/condition, given as a discrete number and unit of measurement
☐	☒	A statement on whether measurements were taken from distinct samples or whether the same sample was measured repeatedly
☐	☒	The statistical test(s) used AND whether they are one- or two-sided<br>*Only common tests should be described solely by name; describe more complex techniques in the Methods section.*
☐	☒	A description of all covariates tested
☐	☒	A description of any assumptions or corrections, such as tests of normality and adjustment for multiple comparisons
☐	☒	A full description of the statistical parameters including central tendency (e.g. means) or other basic estimates (e.g. regression coefficient) AND variation (e.g. standard deviation) or associated estimates of uncertainty (e.g. confidence intervals)
☐	☒	For null hypothesis testing, the test statistic (e.g. F, t, r) with confidence intervals, effect sizes, degrees of freedom and P value noted<br>*Give P values as exact values whenever suitable.*
☒	☐	For Bayesian analysis, information on the choice of priors and Markov chain Monte Carlo settings
☒	☐	For hierarchical and complex designs, identification of the appropriate level for tests and full reporting of outcomes
☐	☒	Estimates of effect sizes (e.g. Cohen's d, Pearson's r), indicating how they were calculated

Our web collection on statistics for biologists contains articles on many of the points above.

Software and code

Policy information about availability of computer code

Data collection	For formating MRI data for BIDs compliance, the following software was used:<br>- Digital Imaging and Communications in Medicine (DICOM) to Brain Imaging Data Structure (BIDS) v2.1.6, found at https://pypi.org/project/dcm2bids/<br><br>No software and/or code were used for the collection of cognitive data.
Data analysis	For MRI analysis the following software was used:<br>- Statistical Parametric Mapping software (SPM12), found at http://www.fil.ion.ucl.ac.uk/spm/software/spm12/)<br>- FreeSurfer v.5.3, found at http://surfer.nmr.mgh.harvard.edu<br>- FSL eddy v5.0.10, found at https://fsl.fmrib.ox.ac.uk/fsl/fslwiki/eddy<br>- DiPy Python v.0.11, found at https://dipy.org<br>- ANTs v1.9, found at http://stnava.github.io/ANTs/<br><br>For gene enrichment analysis, the following software was used:<br>- Cytoscape v3.8.0, found at https://cytoscape.org<br>- gProfiler version e103_eg50_p15_68c0e33, found at https://biit.cs.ut.ee/gprofiler/gost<br><br>For brain network analysis, the following code was used and adapted from:<br>- Brain Connectivity Toolbox (BCT) 03/03/2019, found at: https://sites.google.com/site/bctnet.<br><br>All code for the study can be found at: https://github.com/DanAkarca/generativenetworkmodel, doi:10.5281/zenodo.4762612

All simulations were carried out with custom code written in MATLAB 2019b (Mathworks, 2019b). Visualizations, including cortical maps, were achieved using R (RStudio, 1.2.5033) and Python (v3.7.0).

For manuscripts utilizing custom algorithms or software that are central to the research but not yet described in published literature, software must be made available to editors and reviewers. We strongly encourage code deposition in a community repository (e.g. GitHub). See the Nature Research guidelines for submitting code & software for further information.

Data

Policy information about availability of data

All manuscripts must include a data availability statement. This statement should provide the following information, where applicable:

- Accession codes, unique identifiers, or web links for publicly available datasets
- A list of figures that have associated raw data
- A description of any restrictions on data availability

The datasets supporting the current study have not been deposited in a public repository because of restrictions imposed by NHS ethical approval, but are available from the corresponding author on request. Requests for access can be made by research-based institutions for academic purposes. A response can be expected within at least one week. Unidentifiable simulated data can be found at https://osf.io/h9px4/?view_only=984260dcff444b59819961ece9c724ec.

Field-specific reporting

Please select the one below that is the best fit for your research. If you are not sure, read the appropriate sections before making your selection.

☒ Life sciences ☐ Behavioural & social sciences ☐ Ecological, evolutionary & environmental sciences

For a reference copy of the document with all sections, see nature.com/documents/nr-reporting-summary-flat.pdf

Life sciences study design

All studies must disclose on these points even when the disclosure is negative.

Sample size	The CALM cohort contains N=967 total children (N=805 referred; N=162 unreferred). Of these, N=299 undertook MRI scanning of which N=279 had usable MRI data. N=270 of these had cognitive data available and formed the cohort used in this analysis.
Data exclusions	For the majority of the study, the total CALM cohort of N=270 children were used. For the gene expression analysis, one subject was excluded as they were the only subject to have a positive gamma scalar, causing the subject to be a large outlier in the analysis. This left a sample of N=269 for this section alone.
Replication	Generative network models are innately stochastic. As a result, identical results are unlikely to be obtained upon individual model runs. However, sufficient model attempts (such as our parameter selection protocol; see Methods) allow for replication testing of key statistical findings. All attempts at internal replication, where possible, were successful. Independent replication attempts were carried out twice for each of the two cohorts examined.
Randomization	Randomization is not relevant to the present study. This is because subjects were not allocated into experimental groups.
Blinding	Blinding is not relevant to the present study. This is because subjects were not allocated into experimental groups.

Reporting for specific materials, systems and methods

We require information from authors about some types of materials, experimental systems and methods used in many studies. Here, indicate whether each material, system or method listed is relevant to your study. If you are not sure if a list item applies to your research, read the appropriate section before selecting a response.

Materials & experimental systems

n/a	Involved in the study	
☒	☐	Antibodies
☒	☐	Eukaryotic cell lines
☒	☐	Palaeontology and archaeology
☒	☐	Animals and other organisms
☐	☒	Human research participants
☒	☐	Clinical data
☒	☐	Dual use research of concern

Methods

n/a	Involved in the study	
☒	☐	ChIP-seq
☒	☐	Flow cytometry
☐	☒	MRI-based neuroimaging

Human research participants

Policy information about studies involving human research participants

Population characteristics	Our cohort of CALM children is intentionally heterogenous. Referrers were asked to identify children with cognitive problems related to learning, with primary referral reasons including difficulties with ongoing problems in "language", "attention", "memory", or "learning / poor school progress". Exclusion criteria were uncorrected problems in vision or hearing, English as a second language, or a causative genetic diagnosis. Children could have single, multiple or no formally diagnosed learning difficulty or neurodevelopmental disorder. Our sample of N=270 included in this sample includes 65.9% boys, mean age 117.8 months, age range was 66-223 months and 78 that came from the non-referred comparison sample.<br><br>Our second RED cohort contains n=140 typically developing children (mean age 9.34 years, SD age 1.41 years, range 6.82–12.8 years, 45.7% boys).
Recruitment	Our CALM sample were made up of children referred by practitioners working in specialist educational or clinical services to the Centre for Attention Learning and Memory (CALM), a research clinic at the MRC Cognition and Brain Sciences Unit, University of Cambridge (see Holmes, J. et al. 2019 for full recruitment details).<br><br>Our RED sample were made up of typically developing children who had been recruited from local schools (see Johnson, A. et al. 2020 for full recruitment details).
Ethics oversight	The CALM study protocol was approved by, and data collection proceeded under the permission of, the local NHS Research Ethics Committee (reference: 13/EE/0157).<br><br>The RED study protocol was approved under the permission of the Cambridge Psychology Research Ethics Committee (references: Pre.2013.34; Pre.2015.11; Pre.2018.53).

Note that full information on the approval of the study protocol must also be provided in the manuscript.

Magnetic resonance imaging

Experimental design

Design type	Resting state
Design specifications	10 minutes
Behavioral performance measures	No behavioral performance measures during imaging

Acquisition

Imaging type(s)	Structural and diffusion
Field strength	3T
Sequence & imaging parameters	Magnetic resonance imaging data were acquired at the MRC Cognition and Brain Sciences Unit in Cambridge, on the Siemens 3 T Prisma-fit system (Siemens Healthcare) using a 32-channel quadrature head coil. N=299 CALM children underwent MRI scanning. 20 scans were not useable due to excessive motion (>3 mm movement during the diffusion sequence estimated through FSL eddy), leaving an MRI sample of N=279 children. T1-weighted volume scans were acquired using a whole brain coverage 3D Magnetization Prepared Rapid Acquisition Gradient Echo (MP RAGE) sequence acquired using 1 mm isometric image resolution. Echo time was 2.98ms, and repetition time was 2,250ms. Diffusion scans were acquired using echo-planar diffusion-weighted images with an isotropic set of 68 noncollinear directions, using a weighting factor of b = 1,000s × mm−2, interleaved with 4 T2-weighted (b = 0) volume. Whole brain coverage was obtained with 60 contiguous axial slices and isometric image resolution of 2 mm. Echo time was 90ms and repetition time was 8500ms.
Area of acquisition	Whole brain

Diffusion MRI ☒ Used ☐ Not used

Parameters: Isotropic set of 68 noncollinear directions, b = 1,000s × mm−2

Preprocessing

Preprocessing software	- Statistical Parametric Mapping software (SPM12), found at http://www.fil.ion.ucl.ac.uk/spm/software/spm12/)<br>- FreeSurfer v.5.3, found at http://surfer.nmr.mgh.harvard.edu<br>- FSL eddy v5.0.10, found at https://fsl.fmrib.ox.ac.uk/fsl/fslwiki/eddy<br>- DiPy Python v.0.11, found at https://dipy.org<br>- ANTs v1.9, found at http://stnava.github.io/ANTs/
Normalization	Data were normalized using ANTs v1.9.

Normalization template	Data were registered to the MNI template.
Noise and artifact removal	We applied correction for motion, eddy currents, and field inhomogeneities using the eddy v5.0.10 (https://fsl.fmrib.ox.ac.uk/fsl/fslwiki/eddy).
Volume censoring	Parcipants were removed if they achieved >3 mm movement during the diffusion sequence.

Statistical modeling & inference

Model type and settings	Generative network models were used, simulating individually specific connectomic networks over two distinct parameter spaces across 13 generative models (n=10,000 simulations each), followed by the homophily (matching) generative model with greater specificity (n=50,000 simulations). Networks were seeded with a network common to all participants.
Effect(s) tested	A number of analyses are undertaken, each establishing different aspect of model performance and subsequent co-localisation with gene expression.

Specify type of analysis: ☒ Whole brain ☐ ROI-based ☐ Both

Statistic type for inference (See Eklund et al. 2016)	We use a range of statistics, but broadly rely on p-values and permuted p values.
Correction	For PLS analyses, permutation testing was carried out by permuting the PLS response variable in each subject-wise analysis. The observed X loading (gene loading) was compared to the null distribution to form corrected P values. The same were achieved for latent variable covariances explained (variance explained in both the predictor matrix and response variable).

Models & analysis

n/a	Involved in the study
☒	☐ Functional and/or effective connectivity
☐	☒ Graph analysis
☒	☐ Multivariate modeling or predictive analysis

Graph analysis	Streamline connectomes were binarized as outlined in the Methods.<br>Generative network models were estimated for each subject as outlined in the Methods and Results.<br>The energy equation (2) compares simulated and observed connectomes in terms of degree, clustering coefficient, betweenness centrality and edge length.<br>In Figure 2a-d and 2f, we show group averaged energy landscapes from generative network modeling.<br>In Figure 3a-d we show group level cumulative density functions, and group averaged simulated versus observed measures in the energy equation (2) at the nodal level.<br>In Figure 4 and Supplementary Table 3, we show group level global measures a number of connectome measures.
